# Crystal structure and functional implication of bacterial STING

**DOI:** 10.1038/s41467-021-26583-3

**Published:** 2022-01-10

**Authors:** Tzu-Ping Ko, Yu-Chuan Wang, Chia-Shin Yang, Mei-Hui Hou, Chao-Jung Chen, Yi-Fang Chiu, Yeh Chen

**Affiliations:** 1 Institute of Biological Chemistry, Academia Sinica, Taipei, 115 Taiwan; 2 Institute of New Drug Development, China Medical University, Taichung, 406 Taiwan; 3 Graduate Institute of Integrated Medicine, China Medical University, Taichung, 406 Taiwan; 4 Proteomics Core Laboratory, Department of Medical Research, China Medical University Hospital, Taichung, 404 Taiwan; 5 Research Center for Cancer Biology, China Medical University, Taichung, 406 Taiwan; 6 New Drug Development Center, China Medical University, Taichung, 406 Taiwan

**Keywords:** Structural biology, Bacterial structural biology, X-ray crystallography

## Abstract

Mammalian innate immune sensor STING (STimulator of INterferon Gene) was recently found to originate from bacteria. During phage infection, bacterial STING sense c-di-GMP generated by the CD-NTase (cGAS/DncV-like nucleotidyltransferase) encoded in the same operon and signal suicide commitment as a defense strategy that restricts phage propagation. However, the precise binding mode of c-di-GMP to bacterial STING and the specific recognition mechanism are still elusive. Here, we determine two complex crystal structures of bacterial STING/c-di-GMP, which provide a clear picture of how c-di-GMP is distinguished from other cyclic dinucleotides. The protein-protein interactions further reveal the driving force behind filament formation of bacterial STING. Finally, we group the bacterial STING into two classes based on the conserved motif in β-strand lid, which dictate their ligand specificity and oligomerization mechanism, and propose an evolution-based model that describes the transition from c-di-GMP-dependent signaling in bacteria to 2’3’-cGAMP-dependent signaling in eukaryotes.

## Abstract (web summary)

The bacterial Cyclic-oligonucleotide-Based Anti-phage Signaling System (CBASS) contains a CD-NTase that synthesizes cyclic di- and tri-nucleotides, and bacterial STING proteins recognize c-di-GMP generated by CD-NTase during phage infection and signal the infected bacteria to commit suicide. Here, the authors provide insights into the molecular basis for c-di-GMP recognition of bacterial STING proteins by determining two STING protein crystal structures with bound c-di-GMP from *Prevotella corporis* and *Myroides sp*. ZB35.

## Introduction

Biological conflicts between different organisms and different kingdoms of life have been evolving since ancient time. The arm races never stop especially between bacteria and phages^1–4^. For example, the anti-CRISPR (Clustered Regularly Interspaced Short Palindromic Repeats) proteins in phages combat against the CRISPR-cas system in bacteria^5,6^. The more recently discovered CBASS^7,8^ in the bacterial arsenal consists of a CD-NTase that synthesizes diverse cyclic di- and tri-nucleotide (CDN and CTN) second messengers upon phage infection and an effector protein that binds to the CD-NTase product and induces “programmed cell death” through its various activities^7–9^. In eukaryotes, the cGAS-STING pathway of innate immunity, akin to CBASS, plays critical roles in antiviral, anticancer, and autophagy mechanisms^7,10–12^. During viral infection, the sensor cGAS rapidly recognizes foreign nucleic acid molecules and synthesizes the second messenger 2’3’-cGAMP that activates downstream STING effectors, leading to the expression of interferon-related genes^10^.

A recent study demonstrated that STING proteins indeed originated in bacteria^12^. They are fused with transmembrane or Toll/Interleukin-1 receptor (TIR) domain that confers the anti-phage activity by forming membrane pores or cleaving vital electron carrier NAD^+^^7,13,14^. Morehouse et al. showed that during phage infection, bacterial STING proteins stringently recognize c-di-GMP generated by CD-NTase and signal the infected bacteria to commit suicide as a defense strategy against phage propagation^7,12^. However, the reported 3’,3’-cGAMP complex structure of *Flavobacteriaceae sp*. STING (*Fs*STING) does not distinguish the guanine base from adenine and the rationale for binding preference of c-di-GMP over 3’,3’-cGAMP remains unclear^12^.

To provide additional information about cyclic dinucleotide recognition mechanism by bacterial STING proteins, we expressed, purified, crystallized, and determined the structures of two STING domains from *Prevotella corporis* (*Pc*STING, GenBank ID: KXA32418.1) and *Myroides sp*. ZB35 (*My*STING, IMG Gene ID: 2719779365). The presence of STING-bound c-di-GMP in the crystal allowed detailed analysis of its specific binding mode. To obtain further insights into the function of bacterial STING protein, we separated and purified the oligomerized TIR-STING from the dimers and accessed their NAD^+^ cleavage activity. In addition, the detailed oligomerization mechanism is also unveiled by the symmetry-related STING molecules in our crystal structures.

## Results

### Specific recognition of c-di-GMP by STING

**Fig. 1:**
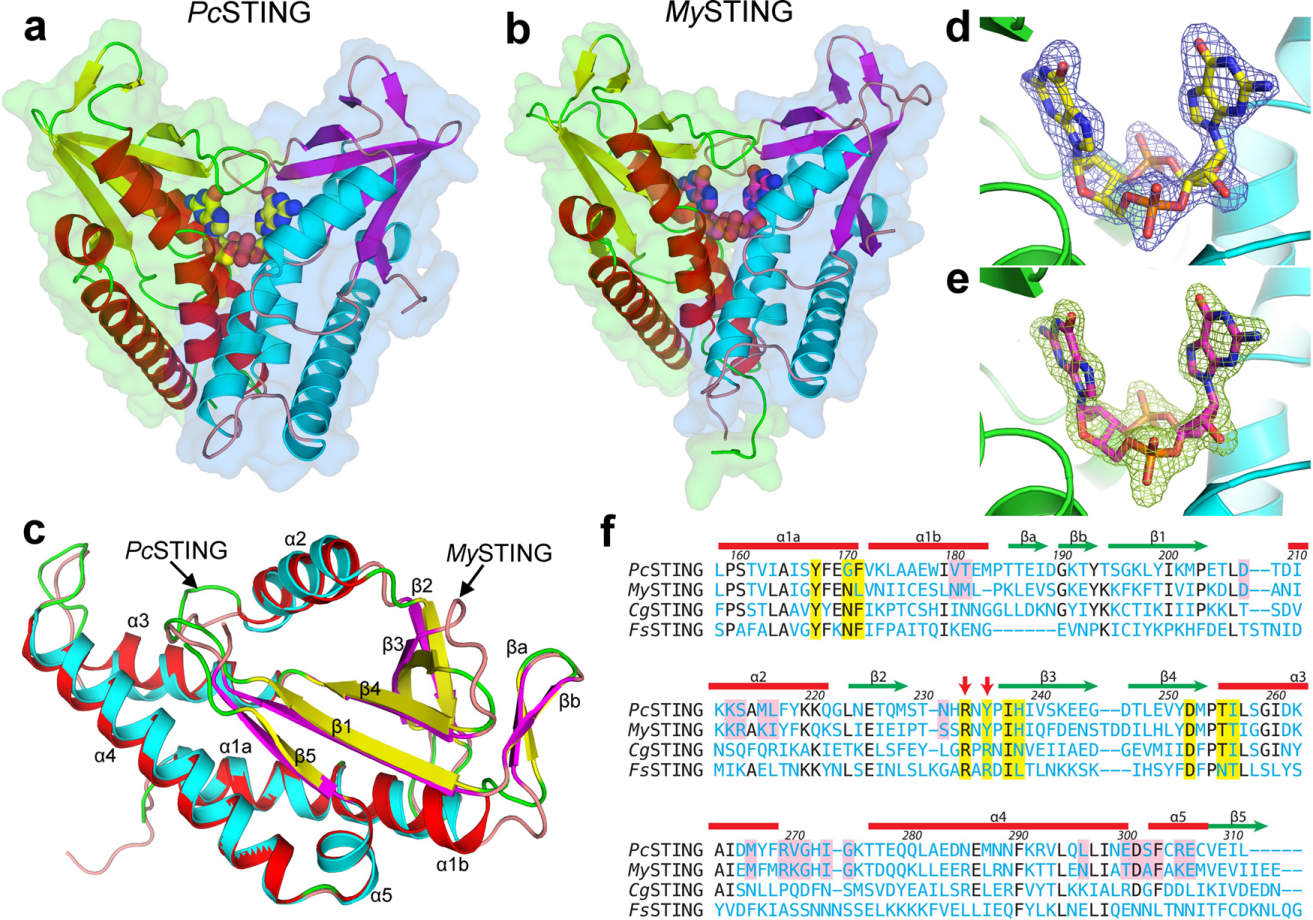
Crystal structures of bacterial STING in complex with c-di-GMP. Surface and cartoon models of dimeric assembly of (**a**) *Pc*STING and (**b**) *My*STING are presented with the two protomers colored differently. The bound c-di-GMP molecule is shown as a space-filled model. **c** The *Pc*STING (colored red–green–yellow) and *My*STING (cyan–pink–magenta) protomers are superimposed and shown as cartoon models with the five α-helices and five β-strands indicated. **d**, **e** The *F*o-*F*c electron-density maps of the bound c-di-GMP molecules, each calculated by using the refined model with the ligand omitted, are contoured at 4-σ level and shown as mesh for the (**d**) *Pc*STING and (**e**) *My*STING crystals. **f** The amino acid sequences of four known bacterial STING proteins are aligned based on their 3D structures. Residues interacting with the bound CDNs are indicated by yellow shade. The conserved RXR or RX(Y/F) motif in the β-strand lid which determines the ligand specificity is indicated by red arrows. The most involved residues in STING oligomerization as identified in this study are indicated by pink shade. *Cg*STING and *Fs*STING refer to the STING proteins from *Capnocytophaga granulosa* and *Flavobacteriaceae sp*.

Initial attempts to solve the *Pc*STING and *My*STING structures by molecular replacement (MR) using the known models of *Fs*STING and *Capnocytophaga granulosa* STING (*Cg*STING) failed, suggesting significant differences between the bacterial STING domains^12^. We turned to prepare SeMet-labeled *Pc*STING and solved the structure at 2.25-Å resolution (Supplementary Table 1). The structure of *My*STING was then solved by MR using the refined model of *Pc*STING (Supplementary Table 2). Both *Pc*STING and *My*STING crystals contained two nearly identical protomers with root-mean-square deviations (RMSDs) of 0.29 Å and 0.64 Å for 158 and 160 matched Cα pairs in the asymmetric unit, forming canonical V-shaped dimers of STING domain (Fig. 1a, b). The overall architectures between *Pc*STING and *My*STING dimers are very similar with a RMSD of 1.09 Å for 272 matched Cα pairs. Each protomer adopts a mixed α/β fold with a central five-stranded β-sheet surrounded by five α-helices and a flanking β-ribbon (Fig. 1c). A previous study showed that the overall dimension of *Fs*STING is smaller than that of human STING (hSTING) (Supplementary Fig. 1a, b)^12^. Our *Pc*STING and *My*STING structures have similar but slightly extended conformation in comparison with *Fs*STING (Supplementary Fig. 1c, d). The dimers show RMSDs of 2.8–3.4 Å from those of *Fs*STING and *Cg*STING, the latter matching mainly on one protomer due to its more open ligand-free conformation. Similar to *Fs*STING, both *Pc*STING and *My*STING dimers contain a bound CDN in the central nucleotide-binding pocket and adopt a closed conformation (Fig. 1a, b). However, the dispositions of helix α2 and loops β3–β4 and α3–α4 vary greatly, as well as the loop lengths of α1–β1 and β2–β3 (Supplementary Fig. 2a, b). Despite the similar overall protein topology, *Pc*STING and *My*STING monomer differs from hSTING by an RMSD of 3.1–3.4 Å for 134–139 matched Cα atoms (Supplementary Fig. 2c, d). Instead of the four-stranded β-sheet lids in metazoan STING, bacterial STING forms a two-stranded β-sheet lid that covers the ligand-binding site. Furthermore, the C-terminal tail (CTT) domain and its preceding helix in mammalian STING are also absent in *Pc*STING and *My*STING (Supplementary Fig. 2c, d)^15^.

**Fig. 2:**
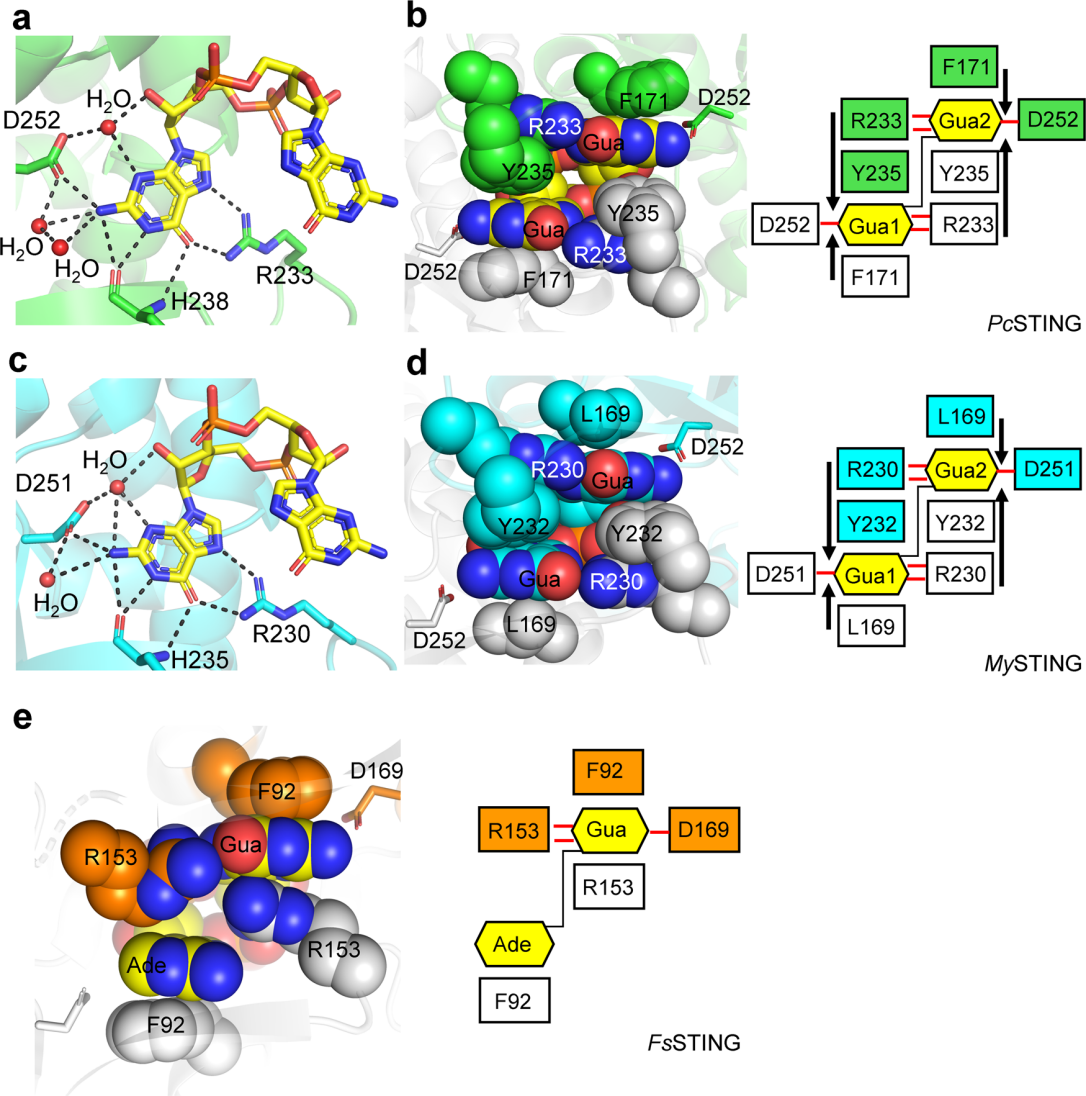
Recognition of c-di-GMP by *Pc*STING/*My*STING and 3’3’-cGAMP by *Fs*STING. **a**, **c** Enlarged view of the ligand-binding pocket of (**a**) *Pc*STING (green) and (**c**) *My*STING (cyan). The hydrogen-bonding network that specifically recognizes the guanine nucleobase of c-di-GMP (yellow) are indicated by a black dashed line. The specificity-determining residues of *Pc*STING, *My*STING, and c-di-GMP molecule are shown as sticks. Water molecules are shown as red spheres. **b**, **d** Left, the four-layer stacking interactions between c-di-GMP and (**b**) *Pc*STING dimer (green and light gray) and (**d**) *My*STING dimer (cyan and light gray); right, schematic representation of the interactions. C-di-GMP and the residues involved in stacking (F171, R233, Y235 in *Pc*STING; L169, R230, Y232 in *My*STING) are shown as spheres. One protomer is colored and the other is white. D252/D251 in *Pc*STING/*My*STING that recognizes N2 of guanine base is shown as sticks. The four-layer stack binding mode is highlighted by black arrows. The H-bonds are indicated by red lines. **e** Left, the stacking interactions between 3’3’-cGAMP (yellow) and *Fs*STING dimer (orange and light gray, PDB: 6WT4); right, schematic representation of the interactions. 3’3’-cGAMP and the residues involved in stacking (F92 and R153 in *Fs*STING) are shown as spheres. D169s in *Fs*STING are shown as sticks. Gua, guanine base; Ade, adenine base.

**Fig. 3:**
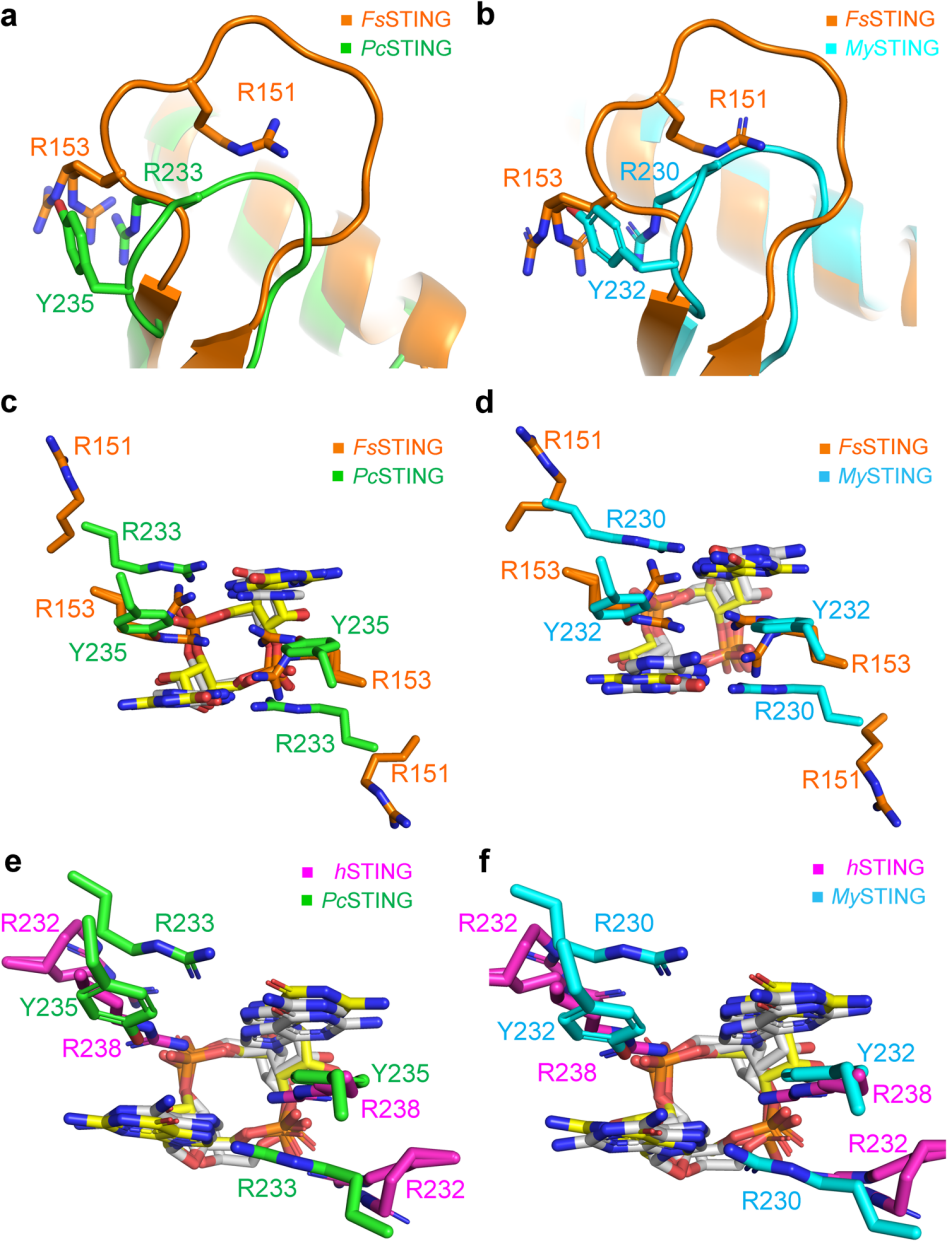
Structural comparison of the β-strand lids and the ligand-binding pockets of bacterial STING and metazoan STING. **a**, **b** Superimposition of the β-strand lids from the 3’,3’-cGAMP-bound *Fs*STING (orange, PDB: 6WT4) and (**a**) *Pc*STING/c-di-GMP complex (green) or (**b**) *My*STING/c-di-GMP complex (cyan). **c**, **d** Enlarged view of the ligand-binding pocket of *Fs*STING superimposed with (**c**) *Pc*STING or (**d**) *My*STING. The specificity-determining residues in β-strand lids are shown as sticks and labeled. C-di-GMP and 3’,3’-cGAMP are shown as yellow and white sticks, respectively. **e**, **f** Enlarged views of the ligand-binding pockets following superimposition of the 2’,3’-cGAMP-bound hSTING (magenta, PDB: 4KSY) with (**e**) *Pc*STING/c-di-GMP complex (green) or (**f**) *My*STING/c-di-GMP complex (cyan). The specificity-determining residues in *Pc*STING, *My*STING and hSTING are shown as sticks and labeled. C-di-GMP and 2’,3’-cGAMP are shown as yellow and white sticks, respectively.

Although no nucleotide was included in the crystallization solution, the strong electron-density unambiguously identified the bound CDN as c-di-GMP (Fig. 1d, e). LC-MS/MS analysis further confirmed the existence of c-di-GMP in the bacterial STING protein samples (Supplementary Fig. 3a–c). Its presence was most likely a result of tight binding and co-purification of the STING proteins with endogenous c-di-GMP from the expression host. The previous study has shown that bacterial STING strongly prefers c-di-GMP over other CDNs^12^. However, the structure of *Fs*STING in complex with 3’3’-cGAMP could not discriminate adenine base from guanine, and the proposed specificity-determining residue D169 of *Fs*STING could not fully explain the specificity for c-di-GMP^12^. Here, our structures demonstrate that the symmetric recognition of single c-di-GMP by dimeric bacterial STING proteins is dictated by the guanidinium group of arginine residue (R233/R230 in *Pc*STING/*My*STING) that recognizes the O6 and N7 atoms of guanine base, the backbone oxygen and amide group (H238/H235 in *Pc*STING/*My*STING) that form three hydrogen bonds to the Watson-Crick edge of guanine base and the aspartate residue (D252/D251 in *Pc*STING/*My*STING) that binds to the N2 atom of guanine base (Figs. 1f and 2a, c). Two or three water molecules further stabilize the interaction by hydrogen bonding to the N1 and N2 positions of guanine base and the free 2’-OH of ribose within c-di-GMP in both *Pc*STING and *My*STING structures (Fig. 2a, c). In addition to the specific H-bonding interaction, each guanine base of c-di-GMP is sandwiched by extensive π–π stacking interaction formed by F171 of one *Pc*STING protomer and Y235 of the other protomer (Fig. 2b). The planar guanidinium group of R233 also stacks with the aromatic ring of Y235, forming a unique Phe/Guanine/Tyr/Arg four-layer stack buttressed by the four H-bond interactions between R233 and c-di-GMP (Fig. 2b). This distinct four-layer stack binding mode of c-di-GMP is also observed in the *My*STING structure (Fig. 2d). Furthermore, the multiple sequence alignment of bacterial STING family proteins demonstrated that the identified specificity-determining arginine residues (R233/R230/R151 in *Pc*STING/*My*STING/*Fs*STING) are conserved (Supplementary Fig. 4). By contrast, R153 in *Fs*STING (equivalent to Y235/Y233 in *Pc*STING/*My*STING), which was proposed to mediate cation–π stacking interaction with guanine base in the previous study, is not conserved and could be replaced by Phe or Tyr in other bacterial STING family proteins (Supplementary Fig. 4)^12,16^. Therefore, it is clear that the four-layer stacking interaction mediated by the binding motif (Phe/Leu)/Gua/(Tyr/Phe)/Arg (e.g., F171/Gua/Y235/R233 in *Pc*STING or L169/Gua/Y232/R230 in *My*STING), together with the arginine residue (R233/R230 in *Pc*STING/*My*STING) that recognizes the Hoogsteen edge of the guanine base, and the aspartate residue (D252/D251 in *Pc*STING/*My*STING) that contacts the N2 position of the guanine base contributes to the stringent specificity for c-di-GMP by bacterial STING proteins. However, unlike *Pc*STING, the recognition of 3’3’-cGAMP by *Fs*STING is asymmetric (Fig. 2e). Inspection of the *Fs*STING structure revealed that the 3’3’-cGAMP and R153 adopt two different conformations each with about 0.5 occupancies (Fig. 2e) One R153 in *Fs*STING dimer form base-stacking interaction with guanine base to avoid the potential clash between the adenine base of 3’3’-cGAMP and the guanidine group of arginine while the other R153 in *Fs*STING dimer form two H-bonds with the guanine base (Fig. 2e). Superimposition of the structure of 3’3’-cGAMP-*Fs*STING with c-di-GMP bound *Pc*STING or *My*STING revealed that the differences in amino acid composition and tertiary structure of β-strand lids probably resulted in the differential recognition of cyclic dinucleotides between *Fs*STING and *Pc*STING/*My*STING (Fig. 3a, b). The conserved specificity-determining arginine residue R151 in *Fs*STING flipped away from the ligand-binding site, so the R153 in *Fs*STING structure could freely adopt two conformations to recognize 3’3’-cGAMP (Fig. 3a–d). By contrast, in *Pc*STING/*My*STING the stacking interaction of R233/R230 and Y235/Y232 locks the conformation of the guanidinium group of R233/R230 into specific hydrogen bonds with the O6 and N7 atoms of c-di-GMP (Fig. 3a–d). To validate the importance of the specificity-determining tyrosine residue of the four-layer stack in c-di-GMP recognition, in vitro binding assays using isothermal titration calorimetry (ITC) were performed. Changing Y232 to arginine in *My*STING indeed reduced the binding affinity to c-di-GMP by nearly 1200-fold in comparison with the wild-type protein (Supplementary Fig. 5a, b). *My*STING showed weak or no binding to 3’3’-cGAMP and c-di-AMP (Supplementary Fig. 5c, d).

Specificity for the symmetric [3’-5’, 3’-5’] phosphodiester bond linkage of 3’3’-c-di-GMP of bacteria STING is achieved by the side chain of conserved Thr residues (T255/T254 in *Pc*STING/*My*STING), Asn residues (N168 in *My*STING) and the main-chain of I256/T255 in *Pc*STING/*My*STING that form hydrogen bonds with phosphate backbone and the free 2’-OH of the ribose within c-di-GMP (Supplementary Fig. 6a, b). The symmetric ligand-recognition mode of bacterial STING thereby excludes its binding to the asymmetric mammalian cGAS product 2’3’-cGAMP. Indeed, superimposition of complex structure of human STING-2’3’-cGAMP with *Pc*STING or *My*STING structure shows that the free 3’-OH of 2’3’-cGAMP would cause serious steric hindrance to the side chain of I256/T255 in *Pc*STING/*My*STING, thus preventing it from binding (Supplementary Fig. 6c, d). Conversely, the intrinsic asymmetric dynamics of metazoan STINGs probably accounts for its preferential binding of 2’3’-cGAMP over other 3’3’-CDNs^17–21^. Previous studies have shown that binding of symmetric 3’3’-c-di-GMP can induce the formation of slightly asymmetric architecture of hSTING dimer, especially in β-strand lid^17–20^. In addition, the asymmetric 2’3’-cGAMP engaged well into the asymmetric ligand-binding pocket of porcine STING dimer, while other symmetric 3’3’-CDNs could not fit well due to steric hindrance or lack of proper interactions^21^. Furthermore, the conserved arginine residues R232 in the β-strand lids of hSTING make contact to the [2’-5’, 3’-5’] phosphodiester bond linkage of 2’3’-cGAMP distinct from the base-specific recognition R233/R230 in *Pc*STING/*My*STING (Fig. 3e, f)^22^. In summary, the structural data presented here fully explained the differential recognition of cyclic dinucleotides by bacterial STING family proteins and suggested that the symmetric/asymmetric ligand-recognition modes of STING proteins from different kingdoms are closely related to their specificity.

### STING oligomerization and TIR activation

**Fig. 4:**
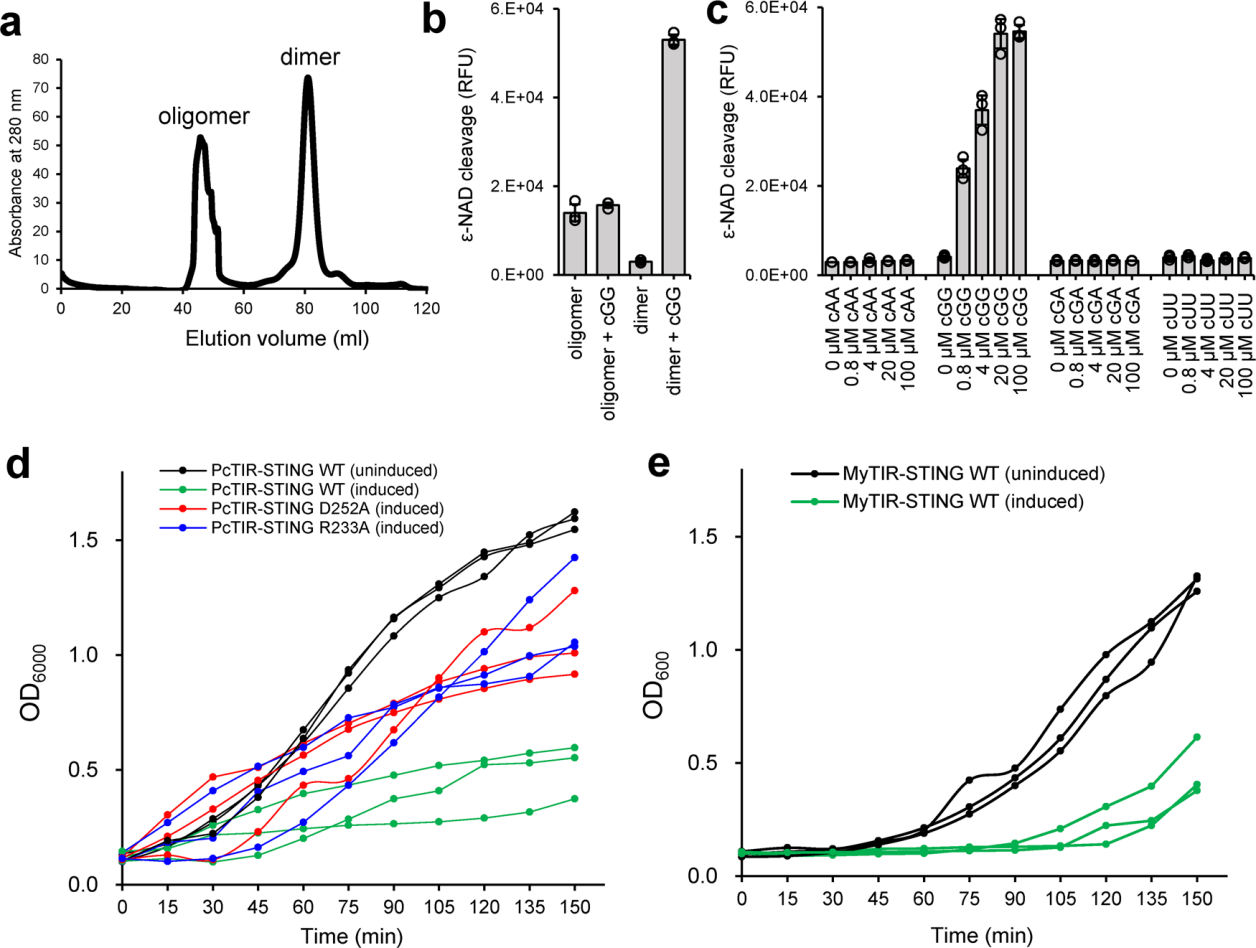
Oligomerization and functional activation of bacterial TIR-STING. **a** Size-exclusion chromatography of *My*TIR-STING proteins. The first peak eluting within void volume was denoted as oligomerized *My*TIR-STING. The second peak was calculated as dimeric *My*TIR-STING. **b** NAD^+^ cleavage activity of the oligomerized or dimerized *My*TIR-STING in the presence or absence of 100 μM c-di-GMP (cGG). **c** NAD^+^ cleavage activity of dimerized *My*TIR-STING with different cyclic dinucleotides (0, 0.8, 4, 20, 100 μM). cAA, cyclic di-AMP; cGA, 3’,3’-cGAMP; cUU, c-di-UMP. The data in (**b**) and (**c**) are shown as mean ± standard deviation for *n* = 3 independent replicates. The effector TIR domain is active only in the presence of c-di-GMP. **d** Growth curves of *E. coli* cells overexpressing wild-type (green line), D252A (red line), or R233A (blue line) mutant *Pc*TIR-STING proteins. The *E. coli* cells carrying wild-type *Pc*TIR-STING without induction (black line) serve as a negative control. Significant bacterial growth inhibition was induced by wild-type *Pc*TIR-STING proteins and largely restored by D252A or R233A mutant which abolish the specificity for c-di-GMP recognition. **e** *My*TIR-STING toxicity analysis using *E. coli* cells producing endogenous c-di-GMP. Induction of the expression of *My*TIR-STING proteins (green line) caused dramatic bacterial growth arrests in host cells compared with the no IPTG induction control (black line). The experiments in (**d**) and (**e**) were performed for *n* = 3 biological replicates and each of them is shown.

Morehouse et al. have shown that c-di-GMP induced the oligomerization of *Sphingobacterium faecium* TIR-STING (*Sf*TIR-STING) into a long filament and activated the NAD^+^ cleavage activity of its TIR domain^12^. However, the functional connection between the oligomerization and activation of TIR domain has not been established. Here, we present solid evidence that the filament formation of bacterial TIR-STING is directly linked to its NADase activation. We have separated and purified the oligomerized *My*TIR-STING proteins from the dimers and accessed their NAD^+^ cleavage activity (Fig. 4a). The results demonstrate that without the addition of c-di-GMP, only oligomerized *My*TIR-STING is active, which probably have incorporated endogenous c-di-GMP from the *E. coli* host (Fig. 4b). Activation of dimeric *My*TIR-STING requires the presence of c-di-GMP, which induces its oligomerization into long filaments (Fig. 4b). It has little effects on the already oligomerized *My*TIR-STING proteins probably due to saturated c-di-GMP binding and imperfect assembly formation, whose NADase activity is also lower than that of freshly activated dimers. The activation effects of other CDNs for the TIR effector domain of bacterial STING proteins were also evaluated by this method. C-di-GMP strongly activated *My*TIR-STING, whereas the addition of up to 100 μM 3’3’-cGAMP, c-di-AMP, or c-di-UMP did not further increase its NADase activity (Fig. 4c and Supplementary Fig. 7a, b). Activation of TIR-STING protein in CBASS immunity system leads to depletion of NAD^+^ and so-called “abortive infections” that restrict bacterial phage reproduction^7^. Indeed, overexpression of wild-type *Pc*TIR-STING or *My*TIR-STING in *E. coli* cells resulted in significant growth inhibition (Fig. 4d, e). Moreover, mutating the specificity-determining residue D252 or R233 in *Pc*TIR-STING relieved the growth inhibition, validating the importance of these two residues in recognition of c-di-GMP (Fig. 4d). In summary, these biochemical data further support the specific activation of the STING-containing CBASS immunity system by c-di-GMP and validate the essential roles of specificity-determining residues identified in this study.

**Fig. 5:**
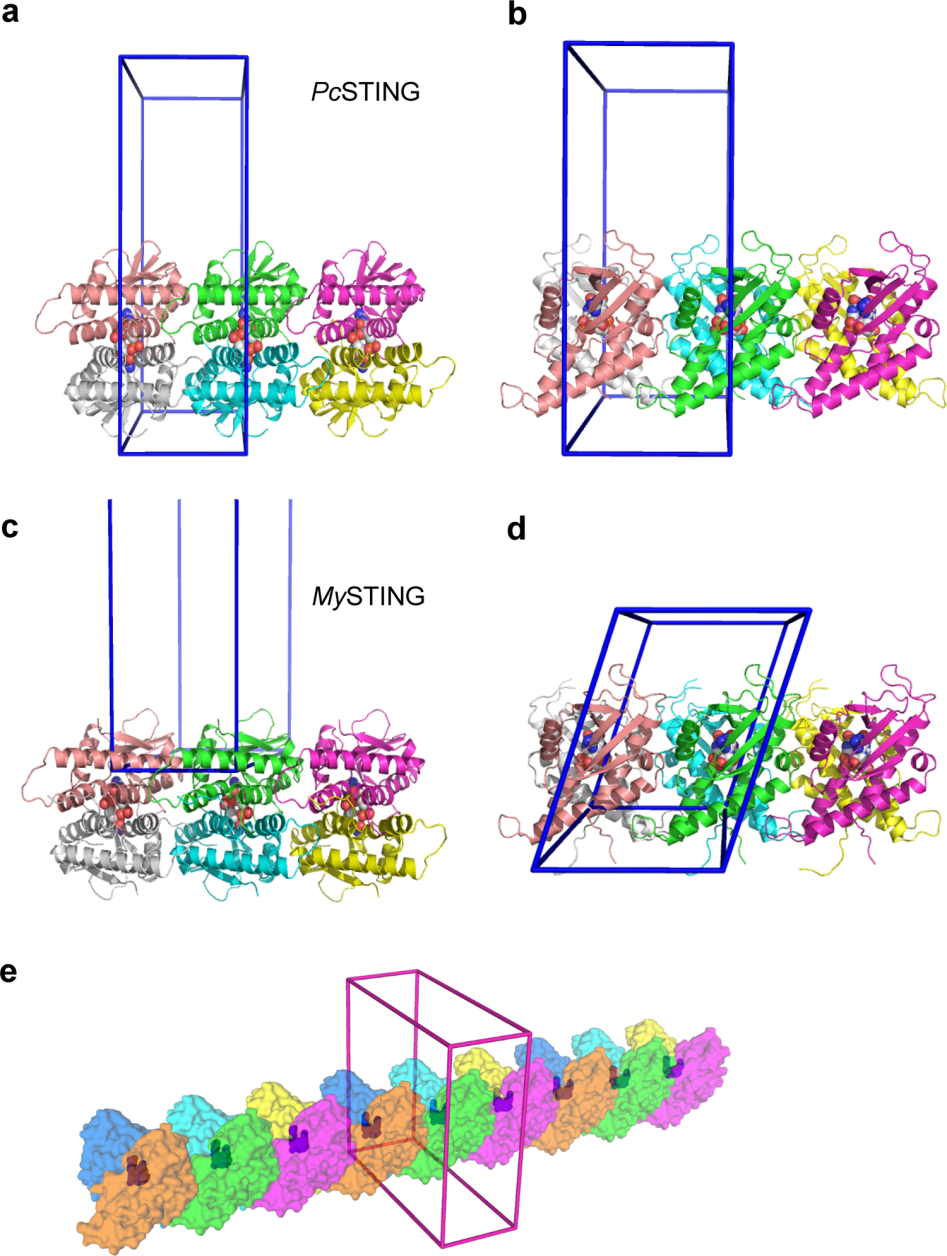
Mode of bacterial STING oligomerization. **a**–**d** Two orthogonal views are shown with three *Pc*STING or *My*STING dimers juxtaposed as seen in the crystals. The protomers are represented by ribbon models in different colors. The bound c-di-GMP molecules are shown as ball models. The unit cells are depicted as blue cages. In (**a**) and (**b**), the *Pc*STING dimers are viewed along the crystallographic *b*- or *a* axis, both with the *c* axis lying horizontally. In (**c**) and (**d**), the *My*STING dimers are viewed with the b-axis lying vertical or pointing upward. Both views of (**c**) and (**d**) have the a axis lying horizontal, and the *My*STING dimers are arranged in a similar way as the *Pc*STING dimers in (**a**) and (**b**). **e** An extended packing diagram for possible filament formation of *Pc*STING dimers is shown with the protomers in alternatively different colors. The bound ligands are also presented by using ball models in blue, which can be seen through the translucent surface diagram of protein models. The unit cell is in magenta.

**Fig. 6:**
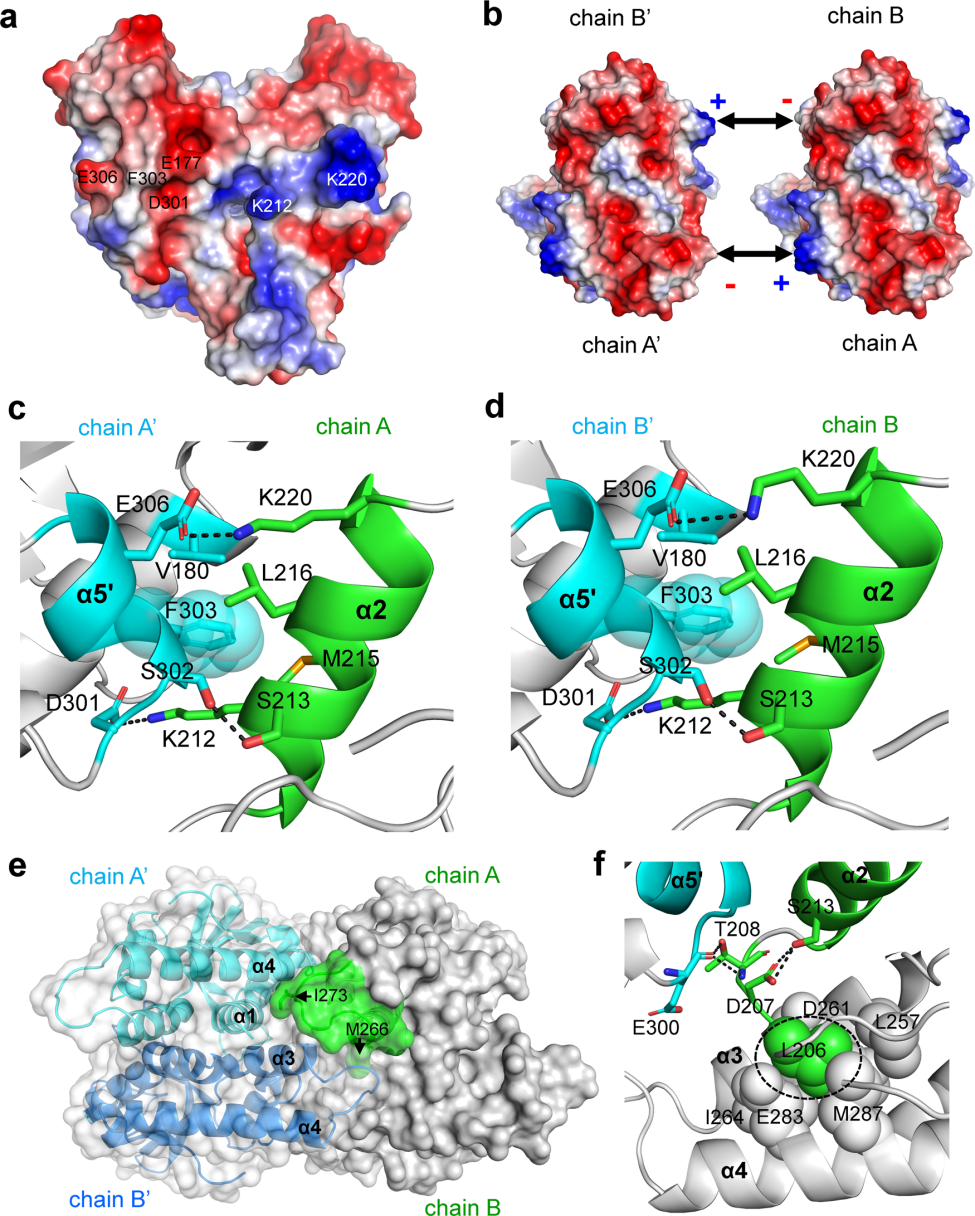
The oligomerization mechanism of *Pc*STING revealed by crystal packing. **a** The electrostatic potential surface of a *Pc*STING dimer. The surfaces are colored in blue for positive potential (10 kcal/mol), red for negative (−10 kcal/mol), and white for neutral. The residues involved in electrostatic interaction and hydrophobic stacking are indicated. **b** Binding scheme of dimer–dimer interaction of *Pc*STING shows that the positively charged patch on one side is complementary to the negatively charged patch on the other side, constituting a basis for filament formation of bacterial STING. **c**, **d** Detailed view of the oligomerization interface between (**c**) chain A and chain A’ or (**d**) chain B and chain B’ of *Pc*STING. The chain A/B of the *Pc*STING dimers in (**b**). The interacting residues are shown as sticks. The H-bond and ionic bonds are indicated in black dashed lines. To emphasize the stacking interaction, the residue F303 is highlighted by showing its sphere model. **e** Surface representation of the *Pc*STING tetramer. The α3-α4 loop (green, residues 267–276) from chain A of one *Pc*STING dimer makes extensive hydrophobic interaction with α1 and α4 helix of chain A’ (cyan) and another α3-α4 loop of chain B’ (blue) of the adjacent *Pc*STING dimer. The side chains of M266 and I273 that participate in oligomerization are indicated by black arrows. **f** The predicted oligomerization interface (residues D119-S123) in *Fs*STING corresponds to the loop region before α2 helix in *Pc*STING. Hydrophobic-interacting residues are shown as spheres and hydrophilic interacting residues are shown as sticks. The conserved leucine residue (L206 in *Pc*STING) that was mutated to arginine and prevented the filament formation in *Sf*STING is highlighted by a black dashed circle. All the α helixes are labeled in bold fonts.

Notably, the oligomerization state is implicated in the crystal structures of both *Pc*STING and *My*STING. Each V-shaped dimer of *Pc*STING associates with its neighbors on both sides, which are symmetry-related by unit-cell translation along the *c* axis (Fig. 5a, b). A similar arrangement of dimers along the *a* axis of *My*STING crystal suggests a common mode of dimer–dimer interaction (Fig. 5c, d). The dimer–dimer interface excludes 1440 Å^2^ surface areas on the *Pc*STING dimer (1420 Å^2^ on *My*STING), comparable to the protomer–protomer interface of 1120 Å^2^ (1500 Å^2^) upon dimer formation. If a continuous filament is formed in this way, each dimer will have ~10% of its surface areas buried and at least 60 amino acid residues involved (Fig. 5e). The oligomerization of *Pc*STING and *My*STING could be attributed to the highly complementary shape and the electrostatic interaction between one side of the *Pc*STING/*My*STING protomer which is rich in positively charged residues and the other side of the *Pc*STING/*My*STING protomer which contains a lot of negatively charged residues (Fig. 6a, b and Supplementary 8a, b). The extensive interfaces include the end of helix α1, the whole helix α2, the loop connecting helix α3 and α4, the end of helix α4, and helix α5 (Fig. 1c). The small helix α5 interacts with helix α2 of the other dimer by both hydrophobic and ionic interactions including two salt-bridges (K212–D301, K220–E306), one hydrogen bond (S213–S302), and the close side-chain packing of F303, K212, M215, and L216 (Fig. 6c). The same contacts are formed on the other side of *Pc*STING dimer (Fig. 6d), and similar interactions are also observed in the *My*STING crystal. For example, the salt-bridging K209 and D300 (equivalent to K212 and D301 in *Pc*STING) together with E175 or an additional N171 contribute two or three ionic bonds that strengthen the filament formation of *My*STING (Supplementary Fig. 8c, d). Moreover, the hydrophobic stacking interactions between the side chains of F302, K209, K212, and I213 are also conserved in *My*STING (Fig. 1f and Supplementary Fig. 8c, d).

The loop connecting helix α3 and α4 (denoted 3–4 loop, residues 267–276) in *Pc*STING intercalated into a groove formed by helix α1 and α4 of protomer A’ and the 3–4 loop of protomer B’ of the other dimer (Fig. 6e). The interactions are mediated mainly by the protein backbone with additional side-chain contacts of I273 and M266 (Fig. 6e). Similar extensive hydrophobic contacts are also observed in the 3–4 loop of *My*STING, with the conserved I272 extended into the groove formed by helix α1 and α4 and M265 interacting with the 3–4 loop of protomer B’ of the other dimer (Fig. 1f and Supplementary Fig. 8e). In summary, the crystal structures of *Pc*STING and *My*STING clearly demonstrate that the interaction of helix α2 with the end of helix α4 and an additional small helix α5 together with the extensive hydrophobic contacts made by the 3–4 loop lock the dimer-to-dimer interface to form a continuous filament. A previous study predicted that the beginning of helix α2, the end of helix α4, and the 3–4 loop are involved in bacterial STING oligomerization^12^. Our study here provides a direct structural account for why mutating these predicted residues in *Sf*STING prevented its oligomerization into long filament^12^. For example, replacement of the 3–4 loop (residues 275–282) with a short linker in *Sf*STING probably disrupt the extensive hydrophobic interaction in the dimer–dimer interface^12^. Interestingly, the 3–4 loop (residues 181–193) is missing in both monomers of the *Fs*STING structure, suggesting that the cGAMP-bound *Fs*STING could not form proper oligomers, or the crystallization condition prevented the protein from oligomerization, and the 3–4 loop was too flexible to be seen in crystal structure^12^. Furthermore, changing A309 (equivalent to F303/F302 in α5 helix of *Pc*STING/*My*STING) to a positively charged arginine in *Sf*STING probably caused repulsion or steric clash in the surrounding residues in helix α2^12^. However, the predicted interface (residues T200-N204 in *Sf*STING) does not directly participate in oligomerization, but instead self-stabilizes the conformation of helix α2 by conserved D207/D204 and helix α3 and α4 by L206/L203 in our *Pc*STING/*My*STING structures (Figs. 1f and 6f and Supplementary Fig. 8f). Mutating L201 of *Sf*STING (equivalent to L206/L203 of *Pc*STING/*My*STING) to arginine may destabilize the tertiary structure, thus hindering its oligomerization. Furthermore, single and double mutants of *My*TIR-STING including K209E, F302A, F302R, K209E/F302A, and K209E/F302R were constructed in order to investigate their effects on the dimer–dimer interface. However, the mutations appeared to result in aggregation of improperly formed c-di-GMP bound *My*TIR-STING oligomers, which might be treated as a waste by the host cell and degraded. Because none of these mutated proteins could be obtained in an intact form for in vitro study, as an alternative, we tested a comparatively stable mutant K209E/F302R for in vivo activity. This double mutant showed nearly no toxicity to *E. coli* cells, which probably resulted from the instability and degradation of the protein (Supplementary Fig. 9a, b).

## Discussion

In this study, we determined high-resolution structures of *Pc*STING and *My*STING in complex with co-purified endogenous c-di-GMP and clearly demonstrated their strong selectivity for c-di-GMP. The structures consistently elucidate the precise binding mode of c-di-GMP to the bacterial STING, which has been further validated by ITC experiments (Fig. 2a–d and Supplementary Fig. 5a). Changing the guanine base to adenine will substitute the O^6^ atom (H-bond acceptor) with the NH_2_ group (H-bond donor), which would result in a significant steric clash with the guanidine group of R233 in *Pc*STING (R230 in *My*STING) and loss of several hydrogen-bonding interactions (Fig. 2a, c). Indeed, the ITC experiments demonstrated that the binding affinity of 3’3’-cGMAP to *My*STING is about 127-fold weaker than that of c-di-GMP (Supplementary Fig. 5a, c). Furthermore, changing c-di-GMP to c-di-AMP completely aborted the interaction with *My*STING (Supplementary Fig. 5d).

**Fig. 7:**
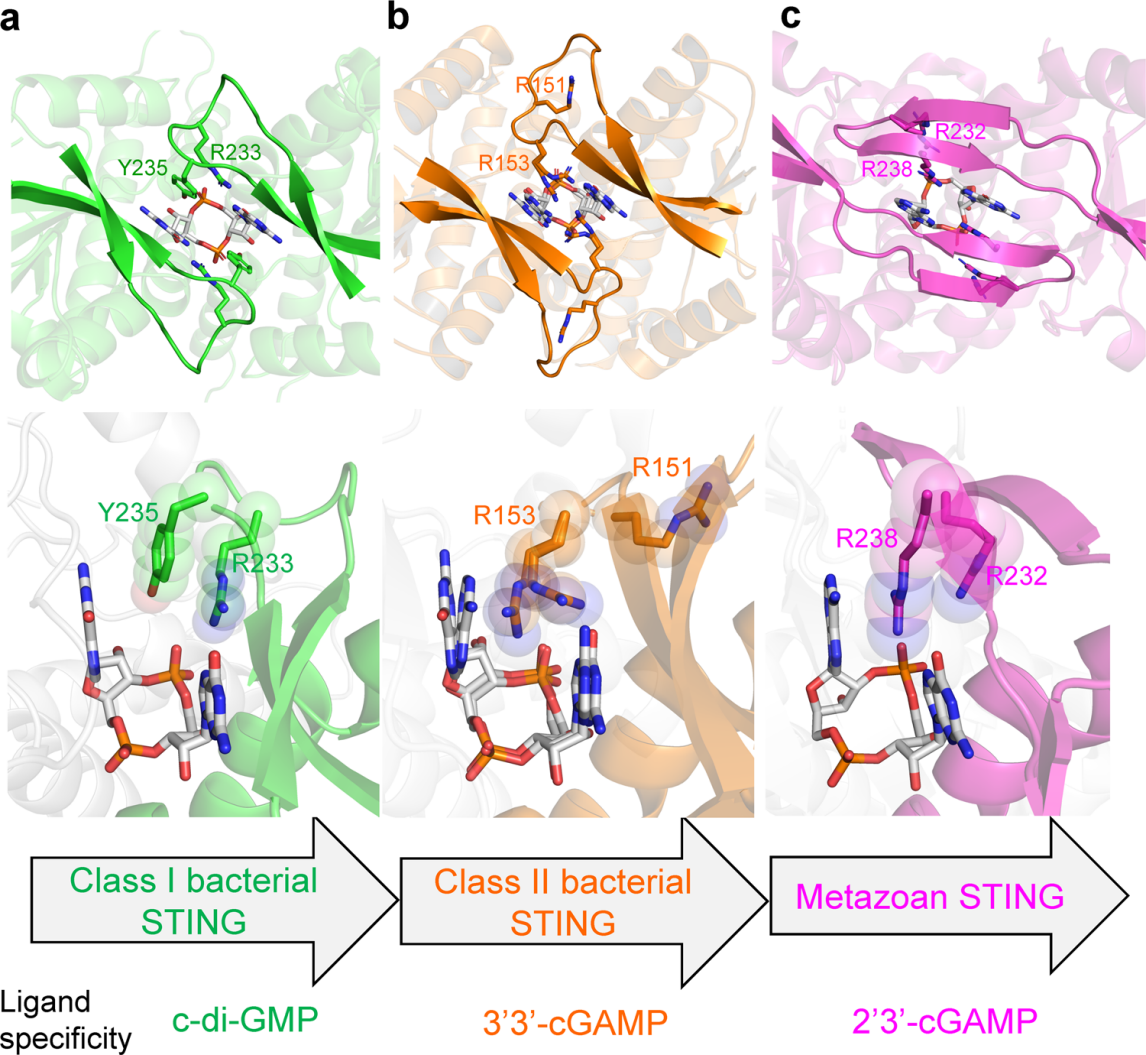
Structural comparison of bacterial and metazoan STING suggests a model for the evolution from c-di-GMP based signaling in prokaryotes to the 2’3’-cGAMP-dependent signaling in eukaryotes. The enlarged view of ligand-binding site and β-strand lid region of (**a**) *Pc*STING-c-di-GMP (green), (**b**) *Fs*STING-3’3’-cGAMP (orange, PDB: 6WT4), and (**c**) human STING-2’3’-cGAMP complexes (magenta, PDB: 4KSY). The cyclic dinucleotide ligands are shown as white sticks. The class I bacterial STING has conserved RX(Y/F) motif (R233-X-Y235 in *Pc*STING) in lid region and specifically recognizes c-di-GMP by four-layer stacking interaction. The class II bacterial STING has conserved RXR motif in the β-strand lid (R151-X-R153 in *Fs*STING), but recognizes 3’3’-cGAMP asymmetrically. The conserved arginine residues of metazoan STING mainly recognize the 2’-5’/3’-5’ phosphodiester linkage of 2’3’-cGAMP instead of the nucleobases. The class II bacterial STING is more closely related to the metazoan STING and is proposed to be acquired into the ancestor of early metazoan.

As mentioned earlier, the R153 of *Fs*STING dimer (equivalent to Y235/Y233 in *Pc*STING/*My*STING) could either nonspecifically base stack with guanine base or make hydrogen bonds to the Hoogsteen edge of guanine base, providing the structural explanation for the recognition of 3’3’-cGAMP by bacterial STING (Fig. 2e). In fact, the bacterial STING family proteins can be grouped into two classes based on the difference in the identified conserved motifs in the β-strand lid, which probably dictate their ligand specificity (Fig. 7). Class I bacterial STING proteins have conserved RX(Y/F) motif in the β-strand lid and utilize the four-layer stack interaction to bind specifically to c-di-GMP as revealed by *Pc*STING and *My*STING structures in this study. Class II bacterial STING proteins have conserved RXR motif in the β-strand lid as exemplified by the solved *Fs*STING structure which preferentially recognized 3’,3’-cGAMP. Interestingly, judging by their positions in the phylogenetic tree, the evolution of bacterial STING proteins has branched into two major categories at an early stage, with Class I on one side and Class II on the other. (Supplementary Fig. 10). According to our classification, *Sf*TIR-STING belongs to Class I STING based on its ^234^RXF^236^ motif and its functional preference for c-di-GMP over 3’3’-cGAMP has been validated by a previous study, which further support our findings^12^. In addition, our structural data and bioinformatic analysis (Fig. 2a, c and Supplementary Fig. 4) also provide an explanation for why mutating the base specificity-determining residue R234 of *Sf*STING (equivalent to R233/R230 to *Pc*STING/*My*STING) to alanine abolish the stringent specificity for c-di-GMP^12^. Although the functional data interpreting the ligand specificity of Class II bacterial STING is currently absent, the only available crystal structure, *Fs*STING, revealed a unique asymmetric recognition of 3’3’-cGAMP, which is distinct from the symmetric four-layer stack binding mode of c-di-GMP by Class I STING. Moreover, the functional role of conserved arginine residues in the β-strand lid of Class II bacterial STING proteins are more closely related to eukaryotic STING, which utilizes two conserved arginine residues (R232 and R238 in hSTING) to recognize the phosphate backbone instead of base-specific interactions (Fig. 7). Therefore, we propose that Class II bacterial STING, which preferentially recognizes 3’3’-cGAMP, is in the evolutionary transition from c-di-GMP-dependent signaling in prokaryote (mediated by Class I STING) to 2’3’-cGAMP-dependent signaling in eukaryote (Fig. 7). The functional role of the conserved arginine residues in β-strand lids changes from base recognition (Class I bacterial STING) to base stacking (Class II bacterial STING) and phosphate backbone recognition (metazoan STING), accounting for the difference in CDN selectivity. This evolution-based model suggests a relationship between STING proteins in different kingdoms of life based on the structural and mechanistic insights provided in this study.

**Fig. 8:**
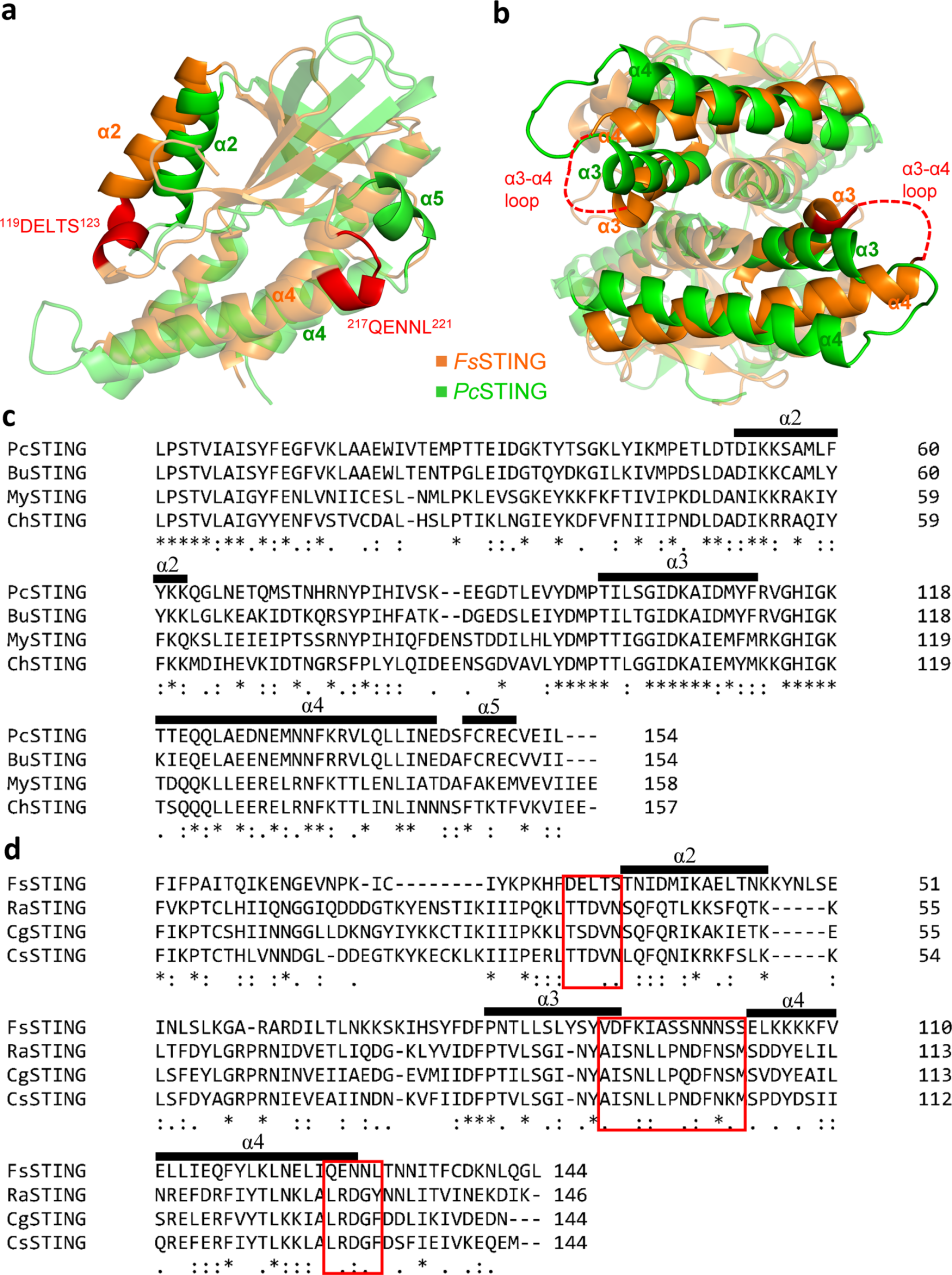
The differences in oligomerization interface between Class I and Class II bacterial STING. **a** The predicted oligomerization interface at the beginning of α2 helix and the end of α4 helix of *Fs*STING (orange, PDB: 6WT4) are indicated and colored in red. By contrast, *Pc*STING (green) protomer uses the α5 helix to interact with the whole α2 helix of the adjacent *Pc*STING protomer. **b** The *Pc*STING and *Fs*STING dimers are superimposed. The orientation of α3 and α4 helix and thereby the predicted α3-α4 loop of *Fs*STING (red dashed lines) are different from those of *Pc*STING. **c** Multiple sequence alignment of four representatives of Class I bacterial STING proteins. **d** Multiple sequence alignment of four representatives of Class II bacterial STING proteins. The predicted oligomerization interfaces in *Fs*STING are highlighted by red frames.

In addition to the differences in the ligand-recognition mode, Class I STING also differ from Class II STING in the tertiary structure and probably the quaternary structure. In comparison with *Fs*STING (Class II), *Pc*STING and *My*STING (Class I) show an additional small helix α5 and a shorter and differently oriented helix α2 (Fig. 8a). As mentioned above, interactions between helix α5 and α2 mediate the oligomerization of Class I STING. However, helix α5 is absent in Class II STING. Instead, the end of helix α4 (^217^QENNL^221^) may interact with the beginning of helix α2 (^119^DELTS^123^) in *Fs*STING based on the model using chicken STING tetramer as a guide^12^ (Fig. 8a). In addition, helices α3 and α4 of *Fs*STING adopt different orientations when compared with *Pc*STING, and thereby the oligomerization mediated by the 3–4 loop may differ between them (Fig. 8b). Moreover, the conserved residues mediating oligomerization in Class I STINGs are distinct from those in Class II STINGs (Fig. 8c, d). For example, the conserved lysine residue in Class I STINGs (K212 in *Pc*STING) at the beginning of helix α2 that forms ionic bonds is absent in Class II STINGs (Fig. 8c, d). The distribution of hydrophilic and hydrophobic residues of helix α2 in Class I STINGs is also different from that in Class II STINGs, which probably leads to different oligomerization interfaces among them (Fig. 8c, d). The previous study has demonstrated that cyclic dinucleotide recognition controls bacterial STING oligomerization and the activation of the adjacent effector domain^12^. Therefore, the differences in primary protein sequences and the tertiary structures suggest that the specific ligand-recognition mode may have coevolved with the oligomerization mechanism, resulting in two distinct classes of bacterial STING proteins (Fig. 7). Indeed, the c-di-GMP or 3’3’-cGAMP induced β-strand lid closure and subsequent conformational changes that oligomerize Class I or Class II STING into active, long filaments are different. C-di-GMP induced the full closure of β-strand lids that cover the CDN binding pocket and form extensive contact mediated by π–π stacking interaction between H238/H235 and Y235/Y232 in *Pc*STING/*My*STING and additional H-bonding interaction between E225 and N234 in *Pc*STING (Supplementary Fig. 11a, b). On the contrary, the 3’3’-cGAMP-bound *Fs*STING showed a loosely packed β-strand lid mediated by only the base-stacking interaction between F92, R153, and the purines of 3’3’-cGAMP instead of direct interaction between residues on strand β2 and β3 of *Fs*STING dimer (Supplementary Fig. 11c)^12^. Superimposition of apo and ligand-bound forms of bacterial STINGs further reveals that structural changes in helices α1 and α4 of *Pc*STING and *Fs*STING are accompanied by induced β-strand lid closure upon c-di-GMP and 3’3’-cGAMP binding, respectively (Supplementary Fig. 12a, b). The conserved phenylalanine residue of α5 helix in Class I STING (F303 in *Pc*STING) is stabilized by the terminal residues of helix α1 (V180 and T181 in *Pc*STING) while the corresponding residue L221 in the loop connecting helix α4 and strand β5 of *Fs*STING is stabilized by the residues F94 and I97 in the middle of helix α1 (Supplementary Fig. 12c, d). Consequently, the differences in cyclic dinucleotide recognition, primary sequence conservation, and the intra- and intermolecular interactions of oligomerization interfaces together result in distinct cyclic dinucleotide-dependent oligomerization between Class I and Class II bacterial STING proteins.

During infection, the innate immunity sensor STING in mammalian cells detect the bacterial CDNs, such as cyclic di-GMP and cyclic di-AMP, as pathogen-associated molecular patterns (PAMPs) and activate the downstream immune responses^23,24^. Until now several human STING structures complexed with c-di-GMP have been solved^17–20,25^. All of them recognize c-di-GMP symmetrically with conserved residues Tyr167 forming π–π stack interaction with guanine nucleobases, which are similar to the base-stacking roles of F92/F171 in *Fs*STING/*Pc*STING structures^12,17–20,25^. In particular, one of the c-di-GMP bound hSTING structures (PDB code: 4F5D) revealed a four-layer stack binding mode for c-di-GMP, consisting of Y167/Gua/R238/Y240 stacking interaction which is similar to the Phe/Gua/Tyr/Arg stacking interaction in *Pc*STING and *My*STING structures identified in this study^17,26^. However, R238 in the four-layer stack of hSTING contacts with the phosphodiester linkage of c-di-GMP instead of the guanine nucleobase as do R233/R230 in *Pc*STING/*My*STING^17^. Adaption of STING proteins for recognizing different cyclic dinucleotides changed the functional role of arginine residues in the β-strand lid, but the common mode of stacking interaction may be conserved during evolution.

## Methods

### Cloning, expression, and purification of bacterial STING proteins

The full-length gene fragments of TIR-STING proteins from *Prevotella corporis* (GenBank ID: KXA32418.1) and *Myroides sp*. ZB35 (IMG Gene ID: 2719779365) were each optimized with *E. coli* codon usage and synthesized by Genomics BioSci & Tech company (Supplementary Table 3). The full-length TIR-STING and C-terminal STING domains were subcloned into pSol-MBP or pSol-SUMO vectors to generate target proteins with N-terminal His_6_-MBP or His_6_-SUMO tag according to the manufacturer’s instructions (Expresso^®^ Solubility and Expression Screening System, Lucigen). Each of the constructs was transformed into *E. coli* BL21(DE3) cells and grown in LB medium at 37 °C for overnight. For large-scale expression, bacterial cultures were induced with 0.2% l-rhamnose until an OD_600_ of 0.6–0.8 was attained and incubated at 16 °C for 16–20 h. For overexpression of TIR-STING proteins, 30 mM nicotinamide was additionally added to the culture mediums to suppress the cell toxicity caused by NAD^+^ cleavage activity of TIR domain. Cell pellets were resuspended in buffer A (50 mM Tris-HCl pH 8.0, 500 mM NaCl, 10% glycerol, 1 mM TCEP, 1 mM phenylmethanesulfonyl fluoride, 5 mM imidazole), lysed using sonication, and centrifuged at 4 °C, 25,000×*g* for 30 min. The clarified supernatant was loaded onto a Ni-NTA column and the recombinant protein was eluted with linear imidazole gradient using ÄKTA™ chromatography system (Cytiva). The N-terminal His_6_-MBP or His_6_-SUMO tag was then removed by incubating the eluted recombinant protein with TEV protease at 4 °C for 24–48 h during dialysis against buffer (25 mM Tris 8.0, 100 mM NaCl, 1 mM DTT, 0.5 mM EDTA, 2% glycerol). After TEV cleavage, the TIR-STING or STING domain proteins were further purified by size-exclusion chromatography using 16/60 Superdex 200 increase column equilibrated with buffer containing 20 mM Tris pH 8.0, 200 mM NaCl, 5% glycerol, 1 mM TCEP. Site-directed mutagenesis of *Pc*TIR-STING, *My*TIR-STING and *My*STING was conducted using *Pfu*Ultra High-Fidelity DNA Polymerase (Agilent). The selenomethionine (SeMet) labeled *Pc*STING was purified with the same protocol of the native protein. All the primers for cloning and site-directed mutagenesis used in this study are summarized in Supplementary Table 4.

### Crystallization, data collection, and structural determination

The optimal protein concentrations for crystallization of *Pc*STING and *My*STING, determined by Pre-Crystallization Test (Hampton), were 6.5 mg/ml and 4 mg/ml, respectively. Initial crystallization screening was conducted at 4 °C using sitting-drop vapor-diffusion method. The crystals of native *Pc*STING and *My*STING were grown in 0.1 M HEPES pH 7.5, 0.2 M calcium chloride dihydrate, 28% v/v PEG 400 and in 0.1 M sodium cacodylate trihydrate pH 6.5, 0.2 M sodium acetate trihydrate, 15% w/v PEG 8000, respectively. The crystals of SeMet-labeled *Pc*STING were grown in 0.1 M sodium cacodylate trihydrate pH 6.5, 0.2 M sodium acetate trihydrate, 18% w/v polyethylene glycol 8000 using the native *Pc*STING crystals as seeds^27^. All the protein crystals were cryoprotected with reservoir solution plus 25% glycerol before flash vitrification in liquid nitrogen. The X-ray diffraction data were collected and processed by using HKL2000 software at the National Synchrotron Radiation Research Center (NSRRC) in Taiwan^28^. Experimental phases of SeMet-labeled *Pc*STING were obtained by multi-wavelength anomalous dispersion (MAD) method using PHENIX software^29^. The three-dimensional model of SeMet-labeled *Pc*STING was then refined and rebuilt iteratively using PHENIX and COOT^29,30^. The structure of *My*STING was determined by molecular replacement using the SeMet-labeled *Pc*STING structure as a searching template. All the data collection, phasing, model building, and refinement statistics are summarized in Supplementary Tables 1 and 2. All the figures that depict three-dimensional structures of protein and ligand were prepared by using PyMOL^31^.

### NAD^+^ cleavage activity assay

To test the catalytic activity of full-length TIR-STING protein, we employed a fluorescent NAD derivative, ε-NAD, for enzyme activity assay^32^. The reaction mixture containing 500 μM ε-NAD, different cyclic dinucleotides (c-di-AMP, c-di-GMP, 3’3’-cGAMP and c-di-UMP) at the indicated concentration in 20 mM HEPES pH 7.5, 100 mM KCl was prepared first. The purified protein at a final concentration of 0.5–1 μM was added to initiate the reaction. The fluorescence signals were monitored continuously for 1 h at the emission wavelength of 410 nm after excitation at 300 nm. The changes of relative fluorescence units at different time points were recorded. For kinetic analysis of *My*TIR-STING protein, the initial velocity of the reaction was determined using the data from the first 300 s.

### Protein toxicity analysis in *E. coli*

The full-length *My*TIR-STING, *Pc*TIR-STING and the mutant constructs were cloned into pET21 vector for generating C-terminal His_6_-tagged proteins or pSol-MBP vector for generating N-terminal MBP-tagged proteins and transformed into *E. coli* BL21(DE3) or C43(DE3) cells. The cells carrying each expression plasmid were first inoculated at 37 °C in LB broth overnight and then diluted by fresh medium to an OD_600_ of about 0.1–0.2. Overexpression of the TIR-STING proteins was induced by the addition of 0.5 mM IPTG (for pET21 vector) or 0.2 % l-rhamnose (for pSol-MBP vector) and 30 mM nicotinamide. The values of OD_600_ were recorded every 15 min for 2.5 h.

### Isothermal titration calorimetry (ITC)

In order to analyze the binding affinity of different CDNs to *My*STING protein and its variant, nano-ITC (TA Instruments) was used. Purified proteins were first dialyzed overnight against assay buffer containing 20 mM Tris pH 8.0, 150 mM NaCl. The CDNs were dissolved in the same buffer. Twenty injections of CDNs (2.5 μl each) at a concentration of 0.15–0.2 mM were sequentially titrated to 8–50 μM wild-type *My*STING or *My*STING_Y232R with a stirring speed of 300 rpm. The heat of dilution resulting from injecting the CDN into the assay buffer was subtracted. The final data were then analyzed using NanoAnalyze software (TA Instruments).

### Bioinformatic analysis

Structure-based sequence alignment of bacterial STING proteins was generated by *Coot*^33^. The phylogenetic tree of bacterial STING proteins was calculated by iTOL^34^.

### LC-MS/MS analysis of cyclic di-GMP

An UHPLC system (Ultimate 3000; Dionex, Germany) equipped with a C18 reversed-phase column (Atlantis T3 C18 3.5 μm 2.1 × 150 mm; Waters) was coupled with a Q-TRAP 6500^+^ mass spectrometer (Sciex, USA) with an orthogonal electrospray ionization (ESI) source. For identification of cyclic di-GMP, the targeted mass was set at m/z 691.1, and major fragment ions of m/z 152.0, *m/z* 248.0 and *m/z* 540.0 were observed with collision energy 32–40 eV. LC gradient elution was applied from 1% mobile phase B (acetonitrile mixed with 0.1% formic acid) to 40% B in the first 15 min at 250 µl/min flow rate and then increased to 99% B within 3 min and held at 99% B for another 3 min. Finally, 1% B was used for 4 min to re-equilibrate the column prior to the next injection. The total run time was 25 min and the mobile phase A consisted of 0.1% formic acid. The ion spray voltage was set at 5.5 kV, and the curtain gas was 25 psi. The ion source gas 1 and 2 were 50 and 60 psi, respectively. The source temperature was set at 550 °C.

### Statistical analysis

The data from NAD^+^ cleavage activity assay shown in Fig. 4b, c and Supplementary 7a, b represent mean ± standard deviation from three independent replicates.

### Reporting summary

Further information on research design is available in the Nature Research Reporting Summary linked to this article.

## Supplementary information

Supplementary Information

Reporting summary

## Data Availability

Data are available within the article and supplementary information. Integrated Microbial Genomes (IMG) database accession and GenBank accession number are listed in the “Methods”. The coordinates and structure factors of *Pc*STING/c-di-GMP and *My*STING/c-di-GMP complexes have been deposited in the Protein Data Bank with accession codes 7EBD and 7EBL. Source data are provided with this paper.

## Source data

Source Data
