# Supplementary material for: Crystal structure and functional implication of bacterial STING
Source: Nat Commun. 2022 Jan 10;13:26. doi: 10.1038/s41467-021-26583-3 (PMC8748872; doi:10.1038/s41467-021-26583-3)
Supplement: Supplementary file 1 — Supplementary Information [file 41467_2021_26583_MOESM1_ESM.pdf]

Supplementary Information

Crystal structure and functional implication of bacterial STING

Tzu-Ping Ko[1,7], Yu-Chuan Wang[2,7], Chia-Shin Yang[2,7], Mei-Hui Hou[2,7], Chao-Jung Chen[3,4], Yi-Fang Chiu[2] and Yeh Chen[2,5,6,*]

[1] Institute of Biological Chemistry, Academia Sinica, Taipei 115, Taiwan
[2] Institute of New Drug Development, China Medical University, Taichung 406, Taiwan
[3] Graduate Institute of Integrated Medicine, China Medical University, Taichung 406, Taiwan
[4] Proteomics Core Laboratory, Department of Medical Research, China Medical University Hospital, Taichung 404, Taiwan
[5] Research Center for Cancer Biology, China Medical University, Taichung 406, Taiwan
[6] New Drug Development Center, China Medical University, Taichung 406, Taiwan
[7]These authors contributed equally to this work: Tzu-Ping Ko, Yu-Chuan Wang, Chia-Shin Yang, Mei-Hui Hou

* To whom correspondence should be addressed. Email: chyeah6599@mail.cmu.edu.tw

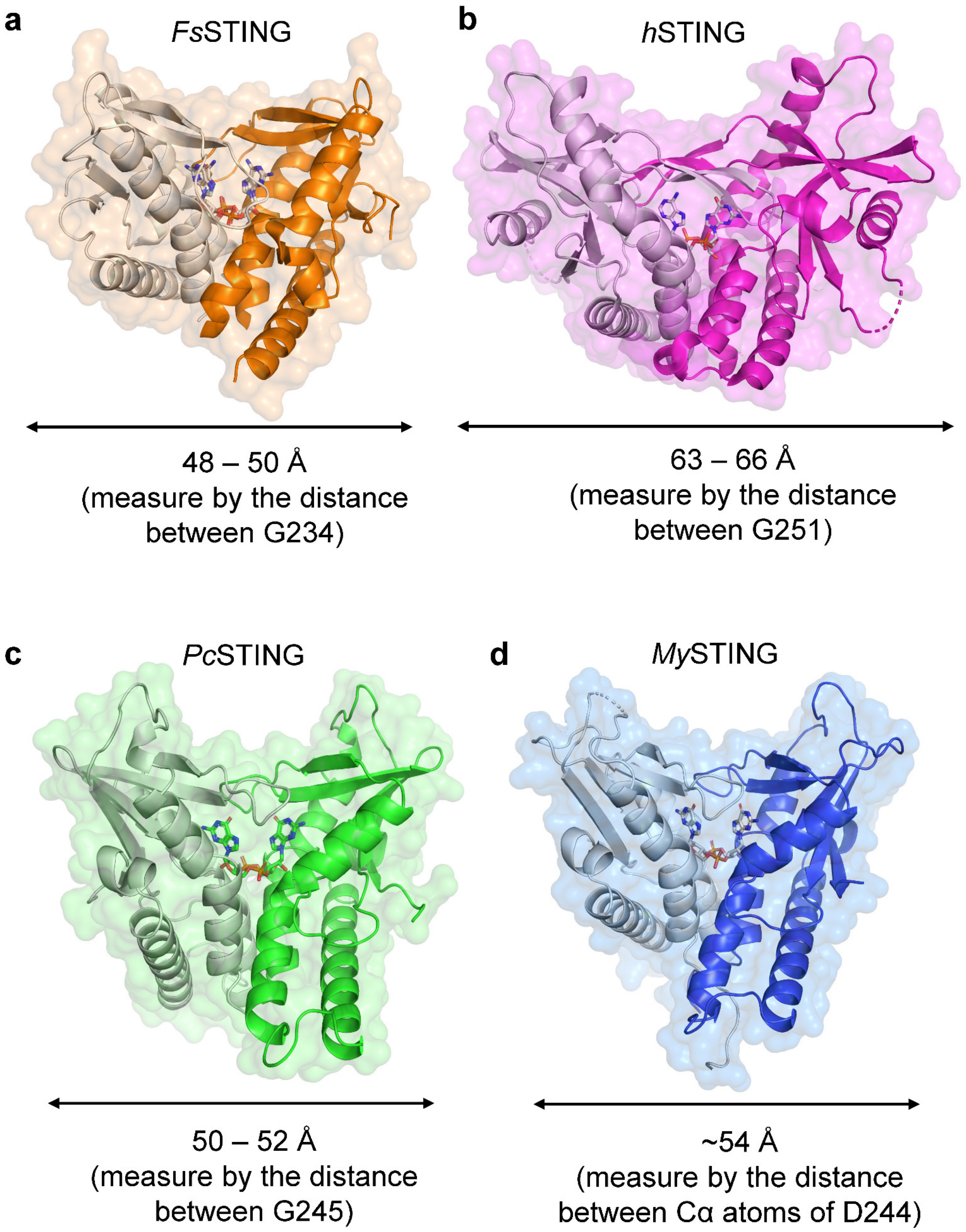

Supplementary Fig.1. Overall architecture of STING family proteins

(a) *Fs*STING-3'3'-cGAMP complex (orange, PDB: 6WT4). (b) *h*STING-2'3'-cGAMP complex (magenta, PDB: 4KSY). (c) *Pc*STING-CDG complex (green, PDB: 7EBD). (d) *My*STING-CDG complex (blue, PDB: 7EBL). The dimension of each STING dimer is indicated.

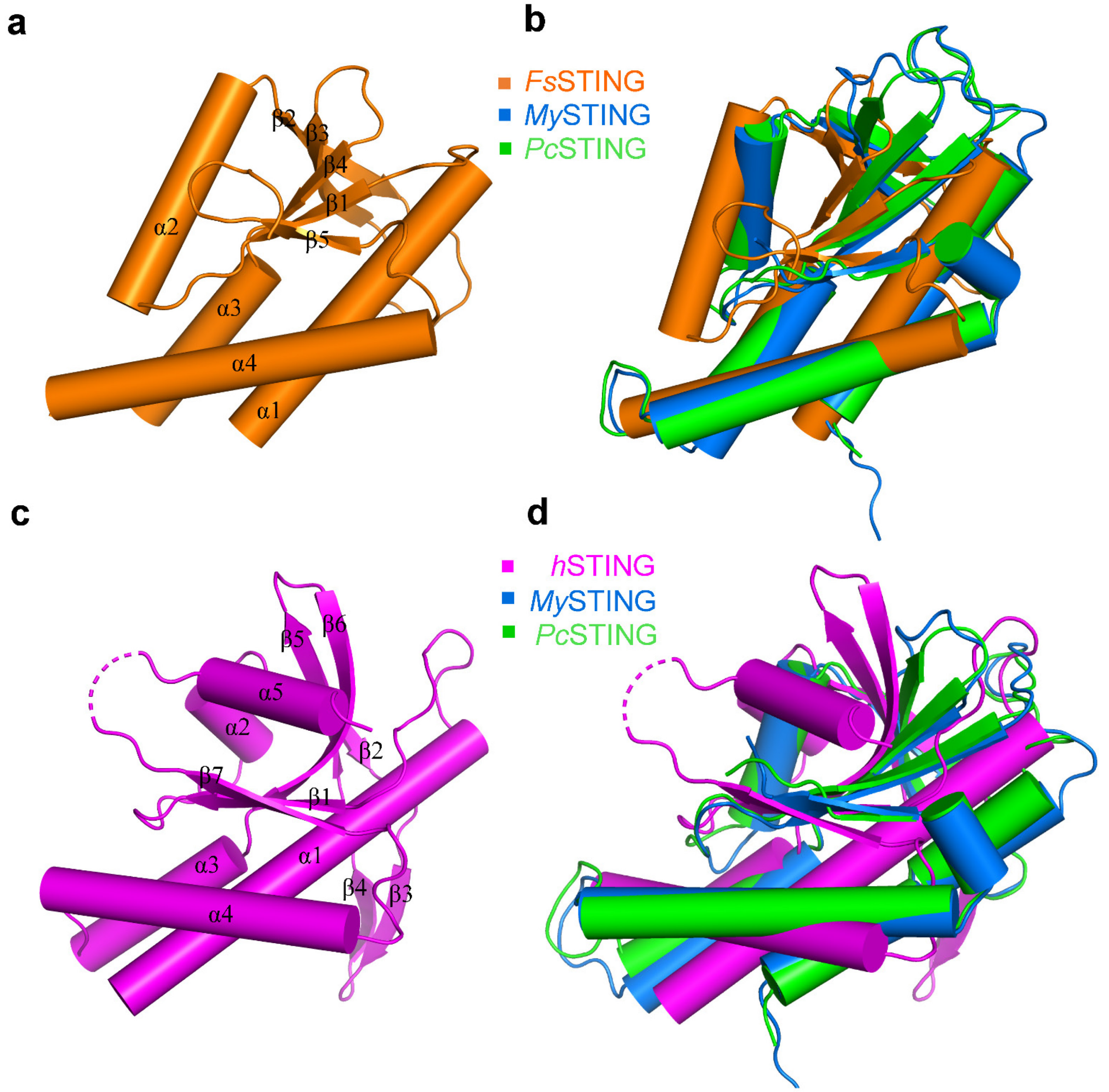

Supplementary Fig. 2. Comparison of the protomeric structure of STING family proteins

(a) Cartoon representation of the protomeric structure of *Fs*STING (orange, PDB: 6WT4) with the four α-helices and five β-strands indicated. (b) The superimposed protomeric structure of *Pc*STING (green) and *My*STING (blue) with *Fs*STING. (c) Cartoon representation of the protomeric structure of *h*STING (pink, PDB: 4KSY) with the five α-helices and seven β-strands indicated. (d) The superimposed protomeric structure of *Pc*STING (green) and *My*STING (blue) with *h*STING.

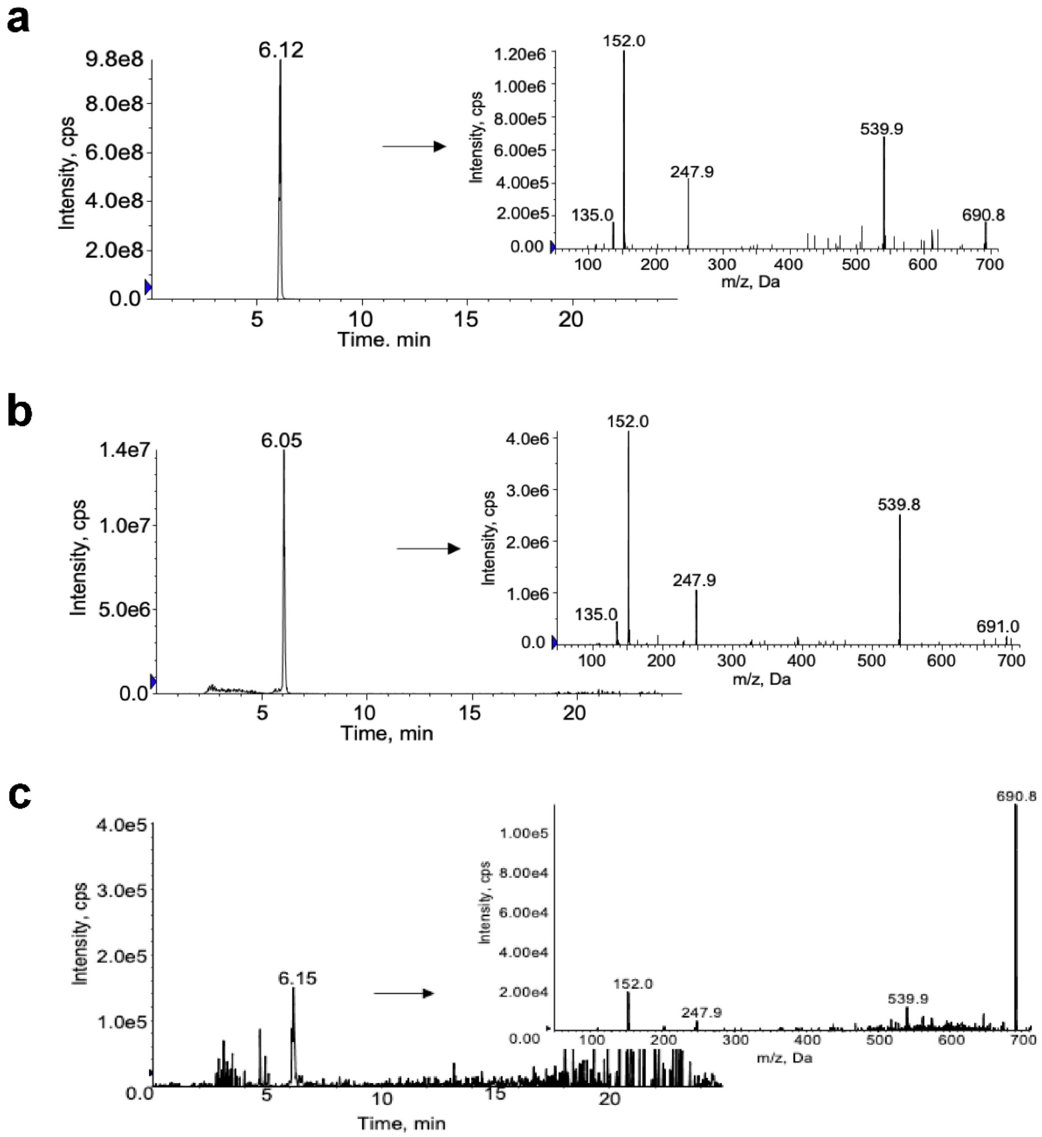

Supplementary Fig. 3. LC-MS/MS analysis of the bacterial STING protein samples

LC-MS/MS analysis using enhanced product ion scan with targeted mass of m/z 691.1. Extracted ion chromatograms (XIC) of a fragment ion (m/z 152.0) and its mass spectrum from (a) a chemical standard of cyclic di-GMP, (b) a sample of *Pc*STING, (c) a sample of *My*STING.

```
FsSTING      FIFPAITQIKENGEV---NPK--------ICIYKPKHFDELTSTNIDMIKAELTNKKYNL      140
PsSTING      LLLPTCKYIIDQDGF-DYESVQ--YRDCIIKVIIPNRLN----TDVNLQFA-QLKRNYQT      220
CsSTING      FIKPTCTHLVNNDGL-DDEGTK--YKECKLKIIIPERLT----TDVNLQFQ-NIKRKFSL      220
RaSTING      FVKPTCLHIIQNGGIQDDDGTK--YENSTIKIIIPQKLT----TDVNSQFQ-TLKKSFQT      223
CgSTING      FIKPTCSHIINNGGLLDKNGYI--YKKCTIKIIIPKKLT----SDVNSQFQ-RIKAKIET      223
SaSTING      LVAPTCKFLIKNKGY-KLNGIQ--YEKFKLKIIIPDKIV----VDPNIAFE-KIKSKLST      219
NdSTING      LIVPICRFIIDNNGF-TKGDTH--YQKCKLNIIVPERIN----QDVNLQFE-KLKGLFTT      220
MsSTING      FIKALCVKIVQDGKL-KIKDAE--YDKCKFQIIIPKKID----EDINLQFE-KIKRETGV      217
TmSTING      FIKYVNEYYINNGGF-IYEGKK--YGDCVFKIMIPETLS----DNLNLQFQ-KEQNRIGV      216
AaSTING      FIKYVCEHFVMNKGF-EFQNKL--HDNCKFKIMIPNTLP----NDLNMAFQ-KIQNKIGV      215
WaSTING      FVKHVCEHYVKNNGF-TYKNNN--YPDCKLRIIIPYKLP----NDLNLAFN-KIQNDIGV      216
SfSTING      FIKRVCEEIHG-SECVELEGKKIKVKSFRVDVVIPETLD----DNGVGNFTTLYNKRYGL      219
DfSTING      FVLPVSRELMQ-SEKRSVEGLN--FADFTLNIVIPDELP----NNF-QDEVIAYLGTHNL      213
MySTING      LVNIICESLNM-LPKLEVSGKE--YKKFKFTIVIPKDLD----ANI-KKRAKIYFKQKSL      220
ChSTING      FVSTVCDALHS-LPTIKLNGIE--YKDFVFNIIIPNDLD----ADI-KRRAQIYFKKMDI      220
DaSTING      FIQLVCDSIGS-GYEMSVNGKN--YTNAKLKIVLPKDLD----SDL-KKKATIYHKKNSF      224
PcSTING      FVKLAAEWIVTEMPTTEIDGKT--YTSGKLYIKMPETLD----TDI-KKSAMLFYKKQGL      223
BuSTING      FVKLAAEWLTENTPGLEIDGTQ--YDKGILKIVMPDSLD----ADI-KKCAMLYYKKLGL      224
LbSTING      FVKLAAEWLVENTPELMINNHK--FNKASLKIVMPESLD----TDI-KRSAMMYYKRHGL      224
             ::                           . :  *  :      :

FsSTING      SEINLSL----KGARARDILTLNK-------KSKIHSYFDFPNTLLSLYSYVDFKIASSN      189
PsSTING      KTVSFQY-----AGRPRNINLDTEIK------DGKLIFIDFPTTLSGINYAISNLLPNDF      269
CsSTING      KKLSFDY-----AGRPRNIEVEAIIN------DNKVFIIDFPTVLSGINYAISNLLPNDF      269
RaSTING      KKLTFDY-----LGRPRNIDVETLIQ------DGKLYVIDFPTVLSGINYAISNLLPNDF      272
CgSTING      KELSFEY-----LGRPRNINVEIIAE------DGEVMIIDFPTILSGINYAISNLLPQDF      272
SaSTING      TKIDIDY-----SGRPRSIHHETIIK------DNIIEFIDFPTIITGINFAISNLLPSEF      268
NdSTING      ENVSFKY-----SGRPRQISVDTQIK------NDTLEFIDFPTIITGINHAISNLLPNDF      269
MsSTING      EQVQIEC-----LGRPRPFQVNSKLL-----ETGELLIIDFPSTLTGINYAIRELLPEEY      267
TmSTING      EKISFGS-----TNRPRNIGVDISIT-----DENKLILIDFPTTLSGINHAISYLLPKEY      266
AaSTING      EKISFGA-----TGRVRNVHVDVKIE------NGQLILLDFPTTLTGIDHAISNLLPNDY      264
WaSTING      DKLSFSA-----FGRPRNVYVDAKVV------GGKLILLDFPTTLTGIDYAISNHLPKDY      265
SfSTING      SKATTCTNPALLGTRGFPFHFKVDPPDANQESPVDIHLLDIPSTLSTIVESLKLYLPSNQ      279
DfSTING      KEMKVET-----VTRKFNFYLDYDYA-----NQESLNLYDLPTTLGALKRAIEMAVPNSY      263
MySTING      IEIEIPT-----SSRNYPIHIQFDENS----TDDILHLYDMPTTIGGIDKAIEMFMRKGH      271
ChSTING      HEVKIDT-----NGRSFPLYLQIDEEN----SGDVAVLYDMPTTLGGIDKAIEMYMKKGH      271
DaSTING      EQLQIET-----KHRQYPLYVSIDS------HYDSLILSDMPTTLNGIDKAIDMYLRVGH      273
PcSTING      NETQMST-----NHRNYPIHIVSKE------EGDTLEVYDMPTILSGIDKAIDMYFRVGH      272
BuSTING      KEAKIDT-----KQRSYPIHFATKD------GEDSLEIYDMPTILTGIDKAIDMYFRVGH      273
LbSTING      EEARIDT-----KHRNYPIHFASKT------EDGILEVYDMPTILTGIDKAIDMYFRVGH      273
                           *   .                    *:*.  :  :   :   .

FsSTING      NNSSELKKKKFVELLIEQFYLKLNELIQENNLTNNIT-FC---DKNLQGL--      235
PsSTING      NSMS-VDYETIIEREIERFVYTLKQLALRNGVD-TLLEIERI---------      309
CsSTING      NKMS-PDYDSIIQREFERFIYTLKKLALRDGFD-SFIEIVKEQEM-------      312
RaSTING      NSMS-DDYELILNREFDRFIYTLNKLALRDGYN-NLITVINEKDIK------      316
CgSTING      NSMS-VDYEAILSRELERFVYTLKKIALRDGFD-DLIKIVDEDN--------      314
SaSTING      NDLT-SDYNLIVERELEKFILTLKQLLLRNNFD-EDVEIIREGTFNKNGANV      318
NdSTING      NKQS-PDYSSILDRELRRFITTLKKLLIRGGFD-EMVNVKRDSEL-------      312
MsSTING      KTFG-DEYENILNRELEKFVYTLNGLIKKNSFD-DFIEIVRV----------      307
TmSTING      REHS-QDYKIILERELNKFIESLEIIFQRNNCN-DFIVIERF----------      306
AaSTING      KNQT-NDYKLILDRELNKFMSTLKKTIEKNEYD-DFVIIERI----------      304
WaSTING      KHQT-DDYNLIIERELNKFIDTLNKILTKNDFD-EFVEIVRS----------      305
SfSTING      VGQD-FDMDYLEMRELENFAKVLKYLIGRNAATKGYVNVLTNVKL-------      323
DfSTING      YGES-ERERVFKKKEMNNFCRALTYLVGNNSITKKKVKITMVDV--------      306
MySTING      IGKT-DQQKLLEERELRNFKTTLENLIATDAFAKEMVEVIIEE---------      313
ChSTING      IGKT-SQQQLLEERELRNFKTTLINLINNNSFTKTFVKVIEE----------      312
DaSTING      IGKS-TEQKLLEERELRNFEMVLRKLVANDAFCKEFVEFITEP---------      315
PcSTING      IGKT-TEQQLAEDNEMNNFKRVLQLLINEDSFCRECVEIL------------      311
BuSTING      IGKK-IEQELAEENEMNNFRRVLQLLINEDAFCRECVVII------------      312
LbSTING      IGKT-NEQKLAEDHEMNNFKRVLQLLINEDAFCRECVEIIEPQP--------      316
                            : .*   *      .        .
```

Supplementary Fig. 4. Multiple sequence alignment of bacterial STING domains in CBASS immunity system

The conserved residues in β-strand lid that constitute the RXR or RX(Y/F) motif which determine the ligand specificity of bacterial STING proteins are indicated by red arrows. The residues involved in oligomerization of bacterial STING proteins identified in this study are indicated by blue arrows. *Ps*STING, *Pedobacter sp.* STING (IMG gene ID: 2524412210); *Cs*STING, *Chryseobacterium soldanellicola* STING (IMG gene ID: 2686056644); *Ra*STING, *Riemerella anatipestifer* STING (IMG gene ID: 2630660691); *Cg*STING, *Capnocytophaga granulosa* STING (IMG gene ID: 2694988876); *Sa*STING, *Siphonobacter aquaeclarae* STING (IMG gene ID: 2623612695); *Nd*STING, *Niabella drilacis* STING (IMG gene ID: 2620490221); *Ms*STING, *Moheibacter sediminis* STING (IMG gene ID: 2718374542); *Tm*STING, *Tenacibaculum maritimum* STING (IMG gene ID: 2568714955); *Aa*STING, *Arenibacter algicola* STING (IMG gene ID: 2574465590); *Wa*STING, *Winogradskyella arenosi* STING (IMG gene ID: 2771484051); *Df*STING, *Dyadobacter fermentans* STING (IMG gene ID: 644929227); *Ch*STING, *Chryseobacterium halperniae* STING (IMG gene ID: 2623152775); *Da*STING, *Dysgonomonas alginatilytica* STING (IMG gene ID: 2731877973); *Bu*STING, *Bacteroides uniformis* STING (IMG gene ID: 2565659645); *Lb*STING, *Lachnospiraceae bacterium* STING (IMG gene ID: 2800731183).

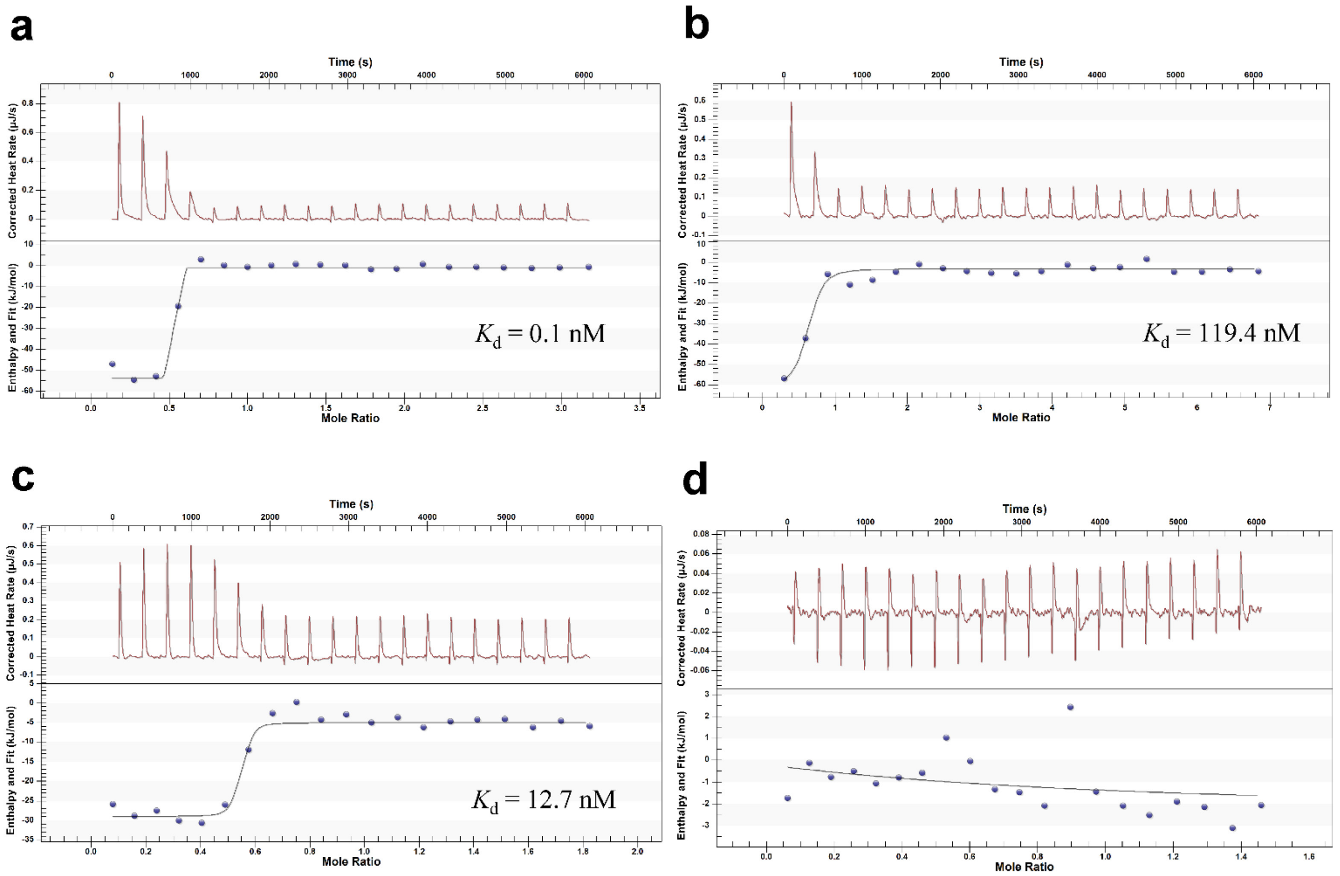

Supplementary Fig. 5. ITC analysis of CDNs binding to bacterial STING proteins

Binding isotherm of (a) c-di-GMP, (c) 3’3’-cGAMP and (d) c-di-AMP to wild-type *My*STING and (b) c-di-GMP to *My*STING_Y232R. Changing of one guanine base to adenine base (c-di-GMP to 3’3’-cGMAP) caused 127-fold decrease in binding affinity to wild-type *My*STING. Changing of c-di-GMP to c-di-AMP abolished the interaction with wild-type *My*STING. Mutating the specificity-determining residue Y232 of *My*STING to arginine reduced the binding affinity of c-di-GMP by 1194-fold compared with wild-type *My*STING.

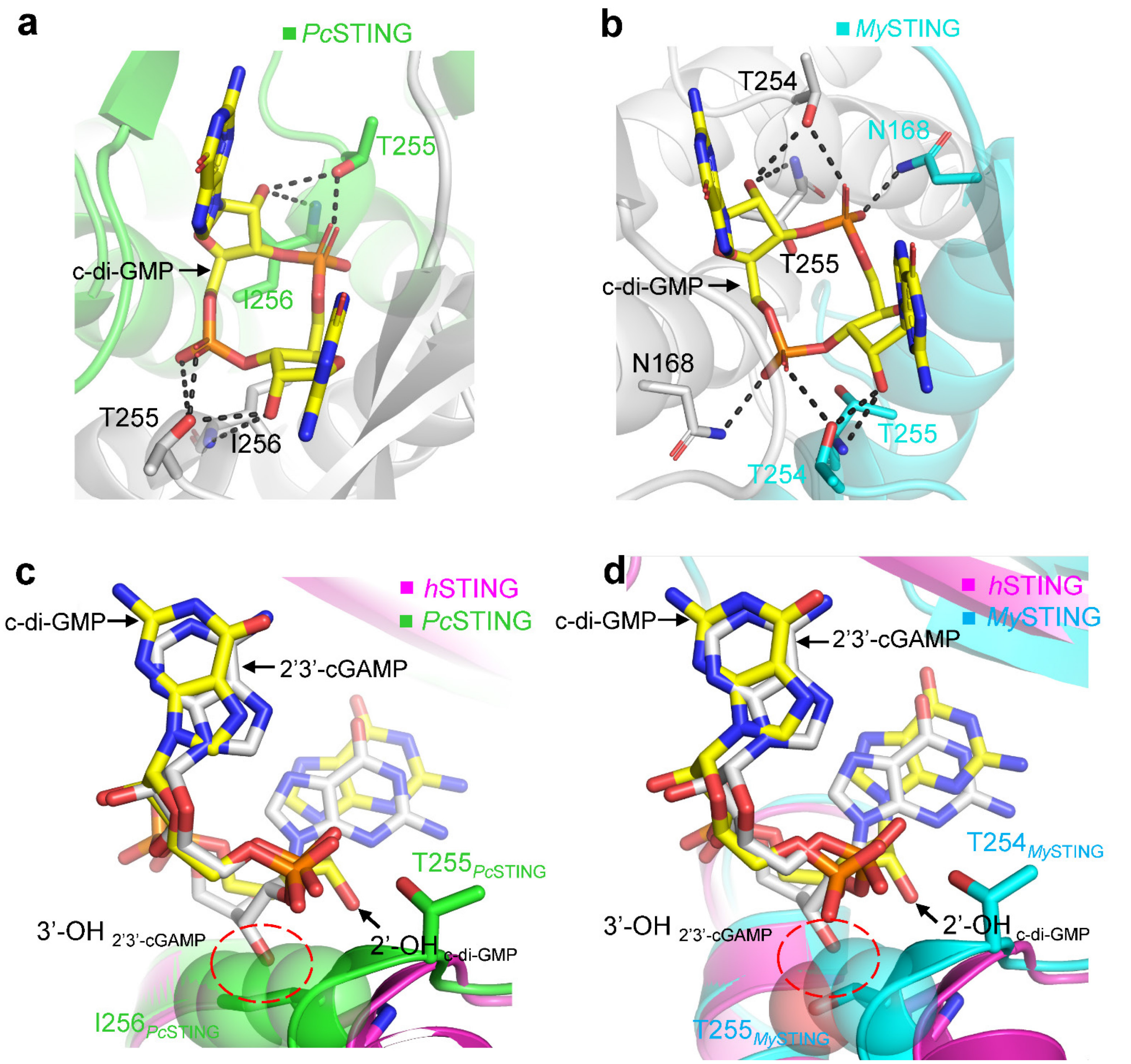

Supplementary Fig. 6. Specificity for [3'-5', 3'-5'] phosphodiester bond linkage of bacteria STING

(a-b) The symmetric recognition of phosphodiester linkage of c-di-GMP by (a) *Pc*STING and (b) *My*STING. The residues involved in hydrogen bonding (black dashed lines) are shown in stick. (c-d) Superimposition of complex structure of human STING/2',3'-cGAMP (blue, PDB: 4KSY) with (c) *Pc*STING/c-di-GMP (green) and (d) *My*STING/c-di-GMP (cyan). C-di-GMP and 2',3'-cGAMP are shown in yellow and white sticks, respectively. The possible steric clash between free 3'-OH of 2',3'-cGAMP and the side-chain of (c) I256 (green sphere) of *Pc*STING or (d) T256 (cyan sphere) of *My*STING is highlighted by red dashed circles.

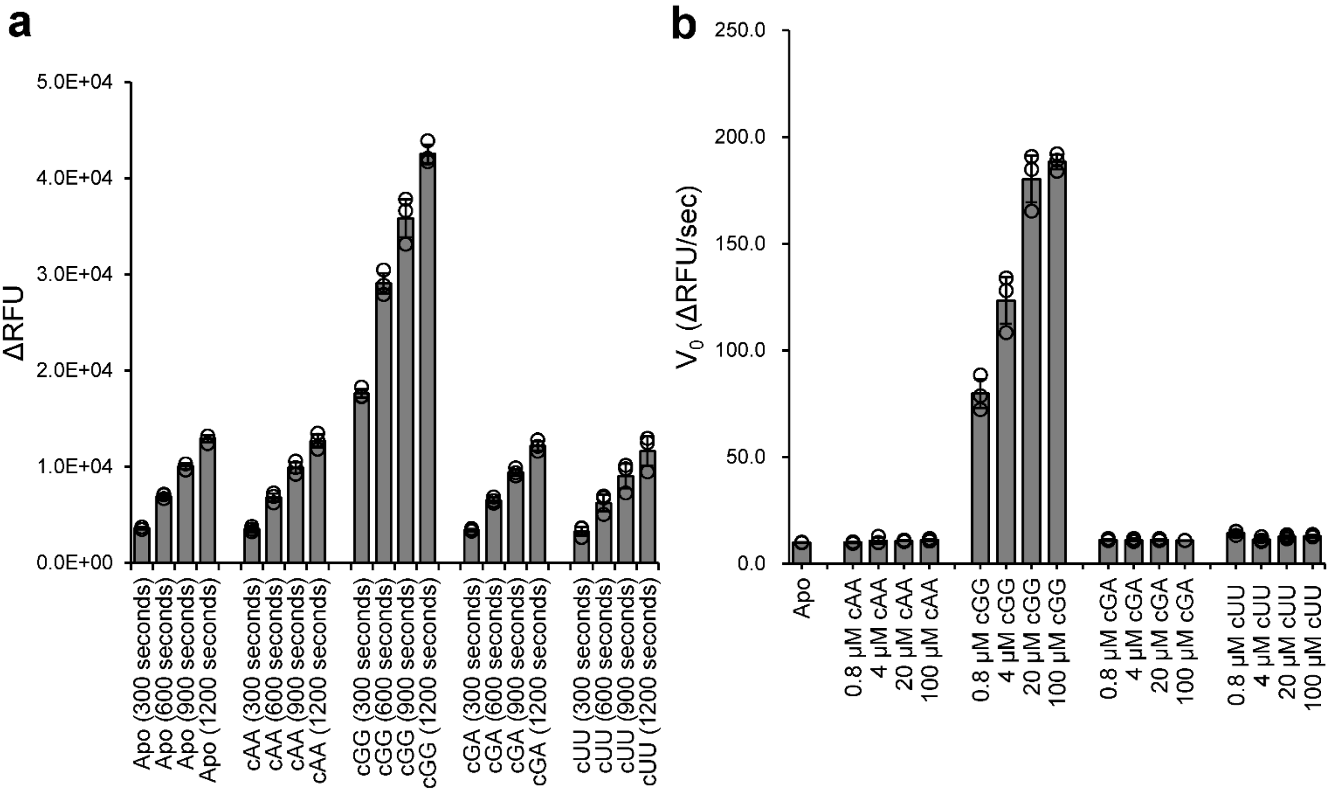

Supplementary Fig. 7. Kinetic analysis of NADase activity of *My*TIR-STING protein

(a) Cleavage of fluorescent NAD^+ analogue, ε-NAD, by *My*TIR-STING protein in different time points (300, 600, 900, 1200 seconds) in the absence or presence of different CDNs at the concentration of 100 μM. Data were represented as mean ± standard deviation for *n* = 3 independent replicates. (b) The initial velocity of NADase activity of *My*TIR-STING protein in the absence or presence of different CDNs at different concentrations (0.8, 4, 20, 100 μM). Data were represented as mean ± standard deviation for *n* = 3 independent replicates.

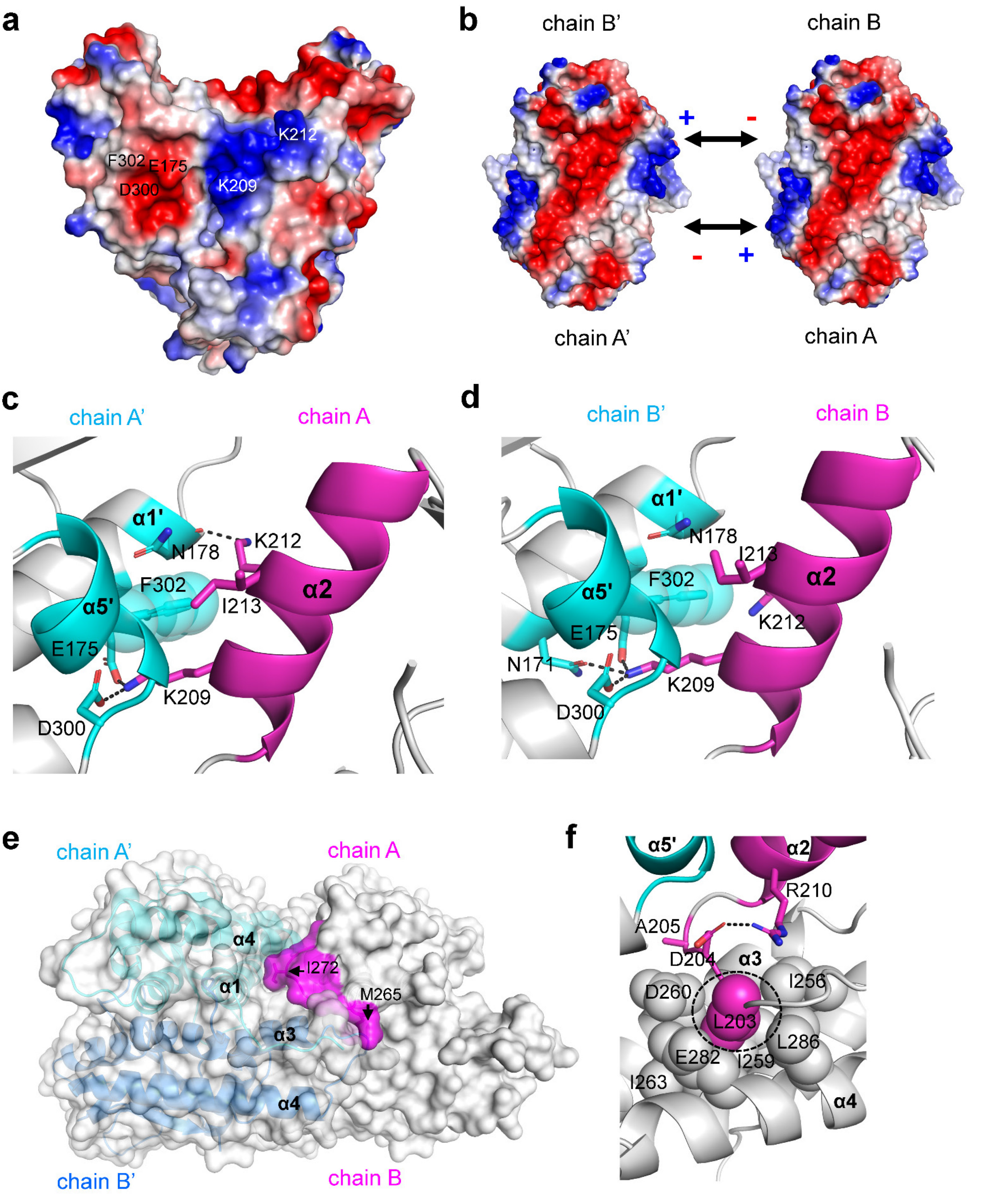

Supplementary Fig. 8. The oligomerization mechanism of *My*STING revealed by crystal packing

(a) The electrostatic potential surface of a *My*STING dimer. The surfaces are colored in blue for

positive potential (10 kcal/mol), red for negative (-10 kcal/mol) and white for neutral. The residues involved in electrostatic interaction and hydrophobic stacking are indicated. (b) Binding scheme of dimer-dimer interaction of *My*STING. (c-d) The detailed view of the oligomerization interface shown in (b) between (c) chain A and chain A' or (d) chain B and chain B' of *My*STING. The chain A/B of *My*STING is colored in magenta while the chain A'/B' are colored in cyan. The interacting residues are shown in sticks. The H-bond and ionic bonds are indicated in black dashed lines. The residue F302 of *My*STING are shown in sphere. (e) Surface representation of the *My*STING tetramer. The α3-α4 loop (magenta, residues 268-274) from chain A of one *My*STING dimer make extensive hydrophobic interaction with α1 and α4 helix of chain A' (cyan) and another α3-α4 loop of chain B' (blue) of the adjacent *My*STING dimer. The sidechain of M265 and I272 participate in oligomerization are indicated by black arrows. (f) The predicted oligomerization interface (residues D119-S123) in *Fs*STING corresponds to the loop region before α2 helix in *My*STING. Hydrophobic interacting residues are shown in sphere and hydrophilic interacting residues are shown in sticks. The conserved leucine residue (L203 in *My*STING) that was mutated to arginine and prevented the filament formation in *Sf*STING is highlighted by black dashed circle. All the α helixes are labeled in bold fonts.

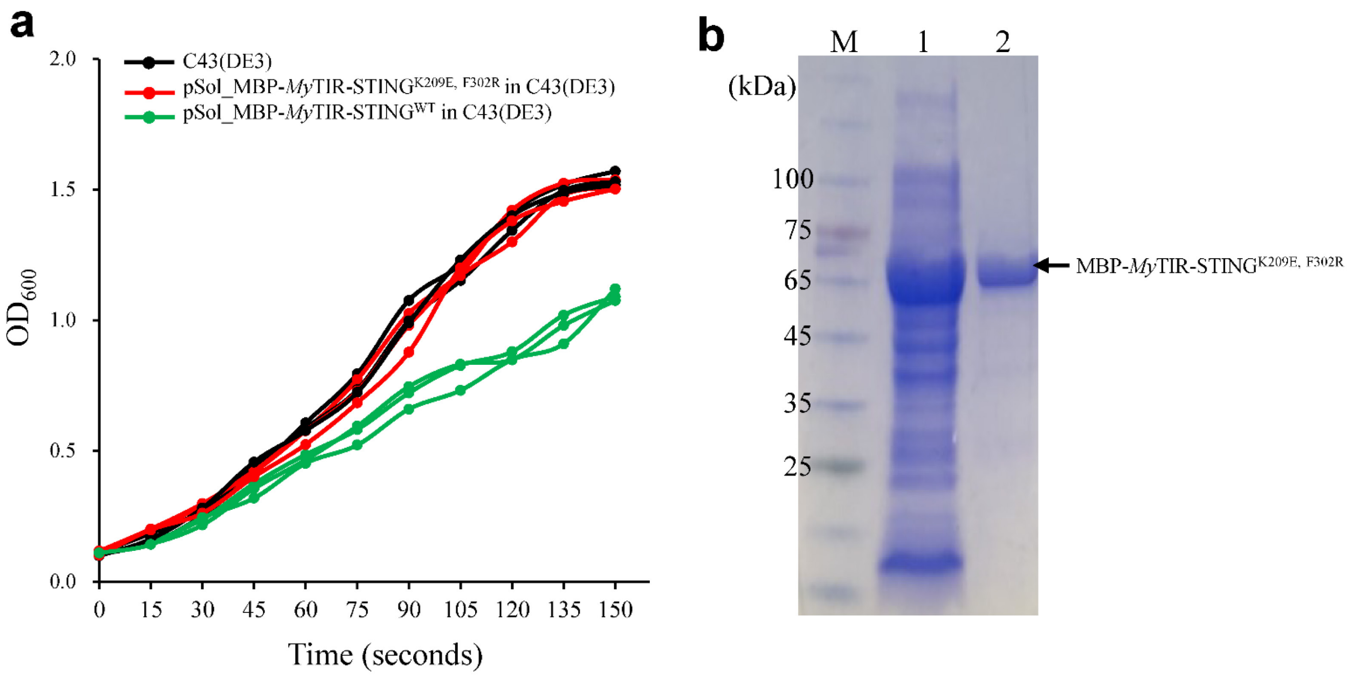

Supplementary Fig. 9. Mutagenesis analysis of dimer-to-dimer interface of *My*TIR-STING
(a) Growth curves of *E. coli* C43(DE3) cells (black line), *E. coli* C43(DE3) cells overexpressing wildtype MBP-*My*TIR-STING (green line) and MBP-*My*TIR-STING^{K209E,F302R} double mutant (red line). The data from three independent experiments are shown. (b) Overexpression and purification of MBP-*My*TIR-STING^{K209E,F302R}. Lane M, Marker. Lane 1, supernatant of *E. coli* lysates overexpressing MBP-*My*TIR-STING^{K209E,F302R}. Lane 2, purified MBP-*My*TIR-STING^{K209E,F302R} by Ni_NTA chromatography. The estimated molecular size of purified MBP-*My*TIR-STING^{K209E,F302R} (~65 kDa) is smaller than the theoretical size of about 78 kDa, probably due to degradation. The unprocessed gel was provided in the Source Data file.

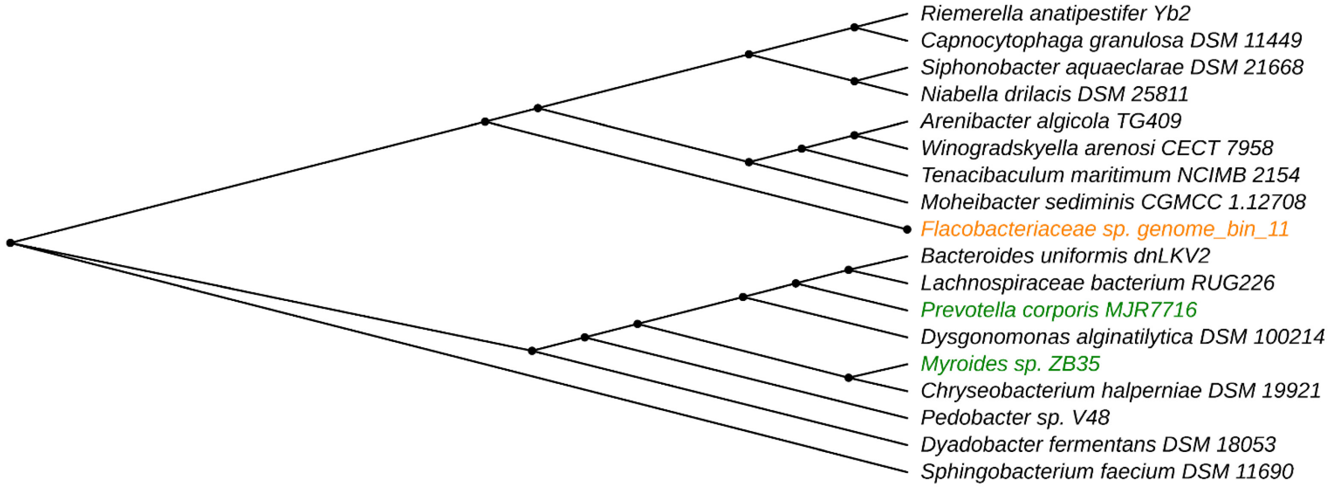

Supplementary Fig. 10. Phylogenetic tree of bacterial STING proteins

Phylogenetic analysis of eighteen representative bacterial STING proteins by iTOL reveal that the divergent evolution of bacterial STING proteins occurs at an early stage and is consistent with the classification in this study, with Class II on the upper group and Class I on the lower group. The currently available crystal structures of Class I and Class II bacterial STING is colored by green and orange, respectively.

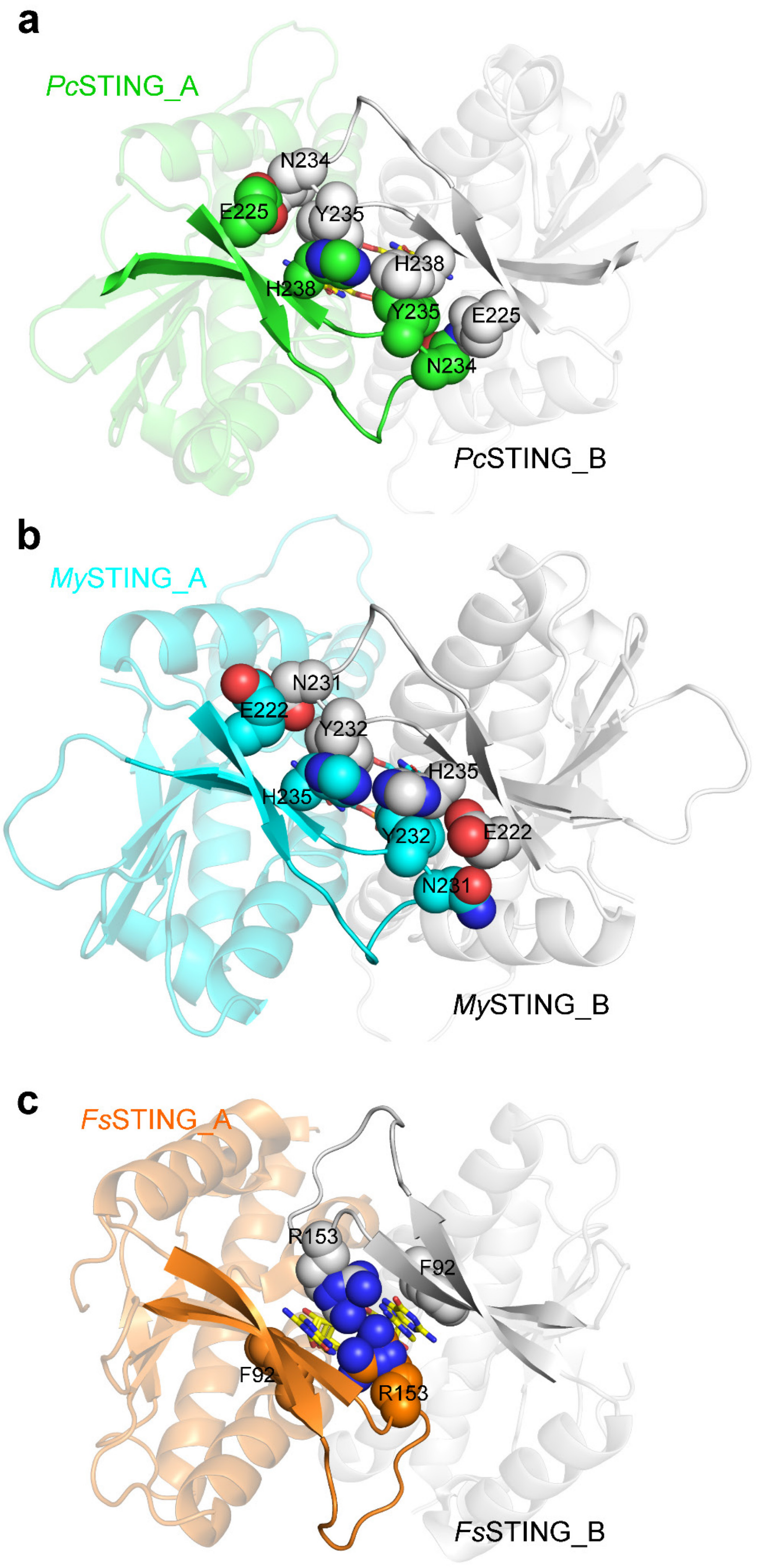

Supplementary Fig. 11. β-strand lid closure of bacterial STING induced by different cyclic

di-nucleotides

(a-b) Fully closed β-strand lids covering the ligand binding pocket induced by binding of c-di-GMP to (a) *Pc*STING (green) and (b) *My*STING (cyan). (c) The 3’,3’-cGAMP molecule stick the β-strand lids together by stacking interaction with R153 and F92 in *Fs*STING (orange). The interacting residues are shown in sphere and labeled. For clarity, protomer A of bacterial STING dimers was colored as indicated above and protomer B was shown in white.

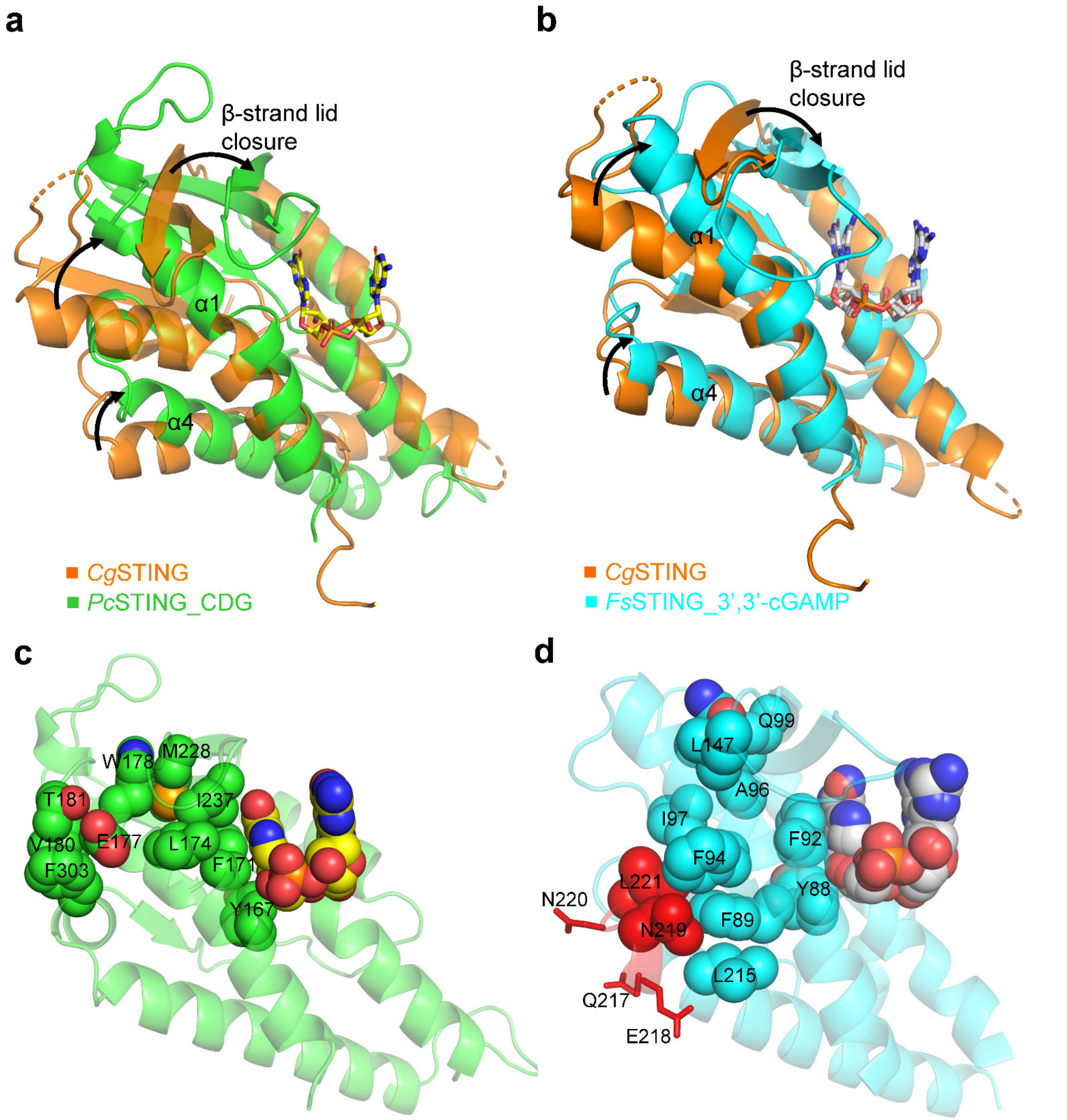

Supplementary Fig. 12. Ligand induced conformational changes in bacterial STING proteins

(a-b) Superimposition of ligand-free *Cg*STING (orange) with (a) CDG bound *Pc*STING (green) or (b) 3'3'-cGAMP bound *Fs*STING (cyan) showed the structural changes within β-strand lid, α1 and α4 helix (black arrows). (c-d) The residues mediated hydrophobic interactions between CDG, β-strand lid, α1 and α4 helix of (c) *Pc*STING and (d) *Fs*STING are indicated and shown in green

and cyan spheres, respectively. CDG and 3’3’-cGAMP are shown in yellow and white spheres, respectively.

Supplementary Table 1. Data collection, phasing, model building, and refinement statistics of SeMet-*Pc*STING crystal.

	Inflection	Peak	High-remote
**Data collection**			
Space group		$P2_12_12$	
Wavelength (Å)	0.97860	0.97836	0.96339
Anomalous term f'	-11.1923	-7.9112	-3.1561
Anomalous term f''	3.6502	6.8486	3.6673
Unit-cell a, b, c (Å)	94.75, 87.28, 35.24	94.53, 87.00, 35.22	94.75, 87.28, 35.24
Resolution range (Å)	30-2.41 (2.50-2.41)	30-2.25 (2.33-2.25)	30-2.42 (2.51-2.42)
Unique reflections	11935 (1171)	14459 (1410)	11795 (1164)
Redundancy	4.7 (4.5)	9.3 (9.0)	4.6 (4.6)
Completeness (%)	99.7 (99.8)	99.8 (100.0)	99.6 (100.0)
Average I / σ(I)	16.05 (2.98)	22.61 (3.80)	16.30 (2.93)
Average $CC_{1/2}$	0.964 (0.882)	0.982 (0.929)	0.961 (0.852)
R_{merge} (%)	9.7 (52.6)	10.6 (61.5)	11.4 (66.0)
R_{pim} (%)	5.0 (27.5)	3.6 (21.5)	3.3 (19.9)
**Phasing and auto-building**			
No. of Se sites found / total		12 / 14	
Figure of merit, Overall score		0.280, 48.1	
Initial / Final No. of amino acids		148 / 209	
Initial / Final map correlation		0.61 / 0.76	
Initial / Final R_{work}, R_{free} (%)		39.7, 42.8 / 29.8, 34.7	
**Structure refinement**			
No. of reflections		14413 (1385)	
Completeness (%)		99.7 (98.4)	
R_{work} for 95% data (%)		18.8 (27.5)	
R_{free} for 5% data (%)		24.9 (32.1)	
RMSD bond lengths (Å)		0.0021	
RMSD bond angles (°)		0.55	
B_{ave} (Å^2) / protein atoms		47.0 / 2532	
B_{ave} (Å^2) / ligand atoms		20.5 / 54	
B_{ave} (Å^2) / water molecules		53.4 / 262	
Ramachandran favored (%)		97.1	
Ramachandran outliers (%)		0.0	
Clashscore		4.29	
MolProbity score		1.48	
**PDB code**		**7EBD**	

Supplementary Table 2. Data collection and refinement statistics of *My*STING crystal.

**Data collection**	
Space group	$P2_1$
Unit-cell a, b, c (Å)	35.25, 98.60, 50.66
β (°)	108.18
Resolution range (Å)	30-2.17 (2.25-2.17)
Unique reflections	17092 (1680)
Redundancy	3.5 (3.0)
Completeness (%)	98.3 (96.9)
Average I / σ(I)	15.74 (3.12)
Average $CC_{1/2}$	0.955 (0.859)
R_{merge} (%)	8.2 (32.0)
R_{pim} (%)	5.2 (21.5)
**Structure refinement**	
No. of reflections	16946 (1583)
Completeness (%)	97.6 (91.9)
R_{work} for 95% data (%)	17.8 (22.4)
R_{free} for 5% data (%)	22.7 (30.7)
RMSD bond lengths (Å)	0.0043
RMSD bond angles (°)	0.81
B_{ave} (Å^2) / protein atoms	34.6 / 2676
B_{ave} (Å^2) / ligand atoms	20.1 / 46
B_{ave} (Å^2) / water molecules	38.6 / 229
Ramachandran favored (%)	97.6
Ramachandran outliers (%)	0.0
Clashscore	3.98
MolProbity score	1.50
**PDB code**	**7EBL**

Supplementary Table 3. The codon-optimized genes used in this study

***Prevotella corporis* TIR-STING (*Pc*TIR-STING)**
ATGAAGCCGCGTATCTTTATTGGTAGCAGCATCGAGGGCCTGGAAGTGGCGAAGCGTATTAAAAG<br>CTTCTTTAGCCCGGACTATGTTTGCTTCCTGTGGACCGATGAGATCTTTAAGAACAACAACAGCTT<br>CCTGGAAACCCTGGTGAAAAGCGCGAGCCTGTTCGACTTTGGTTTCATGGTTTTTAGCGCGGACG<br>ATAAGGCGGTGGTTCGTGAGAAAGGTTTCGACACCCCGCGTGATAACATTCTGTTTGAATATGGCC<br>TGTTCCTGGGTCGTGTGGGCCTGGATCGTGCGTTCGTTATTGCGGAGAAGGACGCGAAAATCCC<br>GACCGATATGCTGGGTATTACCCAGACCCGTTACGAAATCACCATTCGTGACGAGAAGAAAGTGG<br>CGACCGAAAGCCTGGAGGAAGGCCTGATCCTGCTGAAGAAACAAACCGATGAGAACCTGAAACT<br>GGGTCACCTGGGTCTGCTGCCGAGCACCGTTATCGCGATTAGCTATTTTGAAGGTTTCGTGAAGC<br>TGGCGGCGGAGTGGATCGTTACCGAAATGCCGACCACCGAGATTGACGGTAAAACCTACACCAG<br>CGGCAAGCTGTATATCAAAATGCCGGAGACCCTGGACACCGATATTAAGAAAAGCGCGATGCTGTT<br>TTACAAGAAACAGGGCCTGAACGAAACCCAAATGAGCACCAACCACCGTAACTACCCGATCCACA<br>TTGTGAGCAAGGAAGAGGGTGACACCCTGGAAGTTTATGATATGCCGACCATCCTGAGCGGCATT<br>GACAAGGCGATCGATATGTACTTCCGTGTGGGTCACATCGGCAAAACCACCGAGCAGCAACTGGC<br>GGAGGACAACGAAATGAACAACTTTAAACGTGTGCTGCAGCTGCTGATCAACGAAGATAGCTTCT<br>GCCGTGAGTGCGTTGAAATTCTG
***Myroides sp.* ZB35 TIR-STING (*My*TIR-STING)**
ATGAAGCCGCGTATCTTCATTGGTAGCAGCAGCGAAGGCCTGAAAATTGCGGAGTATATC<br>AAGCACAAACTGAGCGAGGAAGAGTTCGACGTGTTTATTTGGACCGACGATATCTTTAAG<br>GCGAACAACAGCGTTCTGGAAACCCTGCTGAAAGAGGCGAGCCTGTTCGATTTTGGTCT<br>GATGATTGCGACCAAGGACGATTACACCACCAAGAAAGACATCGAATTCCAGACCCCGCG<br>TGATAACGTGGTTTTCGAGTTTGGCCTGTTCCTGGGTCGTCTGGGCGTGAACCGTGCGT<br>TTGTTCTGCAAGAAAAAGGTAGCGAGCTGCCGAGCGACCTGTACGGCATTACCGTGCCG<br>CGTTTCGACCTGACCGATAACTTTGAAAACAACTATACCCTGAACAAGGAGATCGATAAGA<br>TCATTAGCAGCATCAAGGAAAAGATTCATCTGGGTGAACTGGGTCTGCTGCCGAGCACC

GTGCTGGCGATCGGTTACTTCGAAAACCTGGTTAACATCATTTGCGAGAGCCTGAACATG
CTGCCGAAACTGGAAGTGAGCGGCAAGGAGTACAAGAAGTTCAAGTTCACCATCGTTATC
CCGAAGGACCTGGATGCGAACATTAAGAAACGTGCGAAAATCTACTTCAAGCAGAAAAGC
CTGATCGAAATTGAGATCCCGACCAGCAGCCGTAACTATCCGATTCACATCCAATTTGACG
AAAACAGCACCGACGATATTCTGCACCTGTACGACATGCCGACCACCATTGGTGGCATCG
ATAAGGCGATTGAGATGTTCATGCGTAAGGGTCACATCGGCAAAACCGACCAGCAAAAGC
TGCTGGAAGAGCGTGAACTGCGTAACTTCAAGACCACCCTGGAGAACCTGATCGCGACC
GATGCGTTTGCGAAAGAAATGGTTGAGGTGATCATTGAAGAG

Supplementary Table 4. Primers used in this study

PCR primers:

**pET-SUMO_*Pc*TIR-STING_F1**	5'-GAACAGATTGGTGGTTCCGGAGGAGGAATGAAGCCGCGTATCTTT-3'
**pET-SUMO_*Pc*TIR-STING_F158**	5'-GAACAGATTGGTGGTTCCGGAGGAGGACTGCCGAGCACCGTTATC-3'
**pET-SUMO_*Pc*TIR-STING_R311**	5'-TACCTAAGCTTGTCTCAGAATTTCAACGCACTCACGGCAG-3'
**pSol_*My*TIR-STING_F1**	5'-AATCTGTACTTCCAGGGTTCCGGAGGAGGAATGAAGCCGCGTATCTTC-3'
**pSol_*My*TIR-STING_F148**	5'-AATCTGTACTTCCAGGGTATTCATCTGGGTGAACTG
**pSol_*My*TIR-STING_R313**	5'-GTGGCGGCCGCTCTATTACTCTTCAATGATCACCTCAAC-3'

The sequences underline in red are additional linker region (Ser-Gly-Gly-Gly) added to the N-terminal of target protein.

The sequences underline in green are required for cloning into the pET-SUMO Vector.

The sequences underline in blue are required for cloning into the pSol Vectors.

Mutagenic primers:

***Pc*TIR-STING_D252A**	
Forward primer	ACACCCTGGAAGTTTATGCTATGCCGACCATCCTGAG
Reverse primer	CTCAGGATGGTCGGCATAGCATAAACTTCCAGGGTGT
***Pc*TIR-STING_R233A**	
Forward primer	CAAATGAGCACCAACCACGCTAACTACCCGATCCACAT
Reverse primer	ATGTGGATCGGGTAGTTAGCGTGGTTGGTGCTCATTTG

***My*TIR-STING_Y232R**	
Forward primer	CCCGACCAGCAGCCGTAACCGTCCGATTCACATCCAATT
Reverse primer	AATTGGATGTGAATCGGACGGTTACGGCTGCTGGTCGGG
***My*TIR-STING_K209E**	
Forward primer	CCTGGATGCGAACATTAAGGAACGTGCGAAAATCTACTTC
Reverse primer	GAAGTAGATTTTCGCACGTTCCTTAATGTTCGCATCCAGG
***My*TIR-STING_F302A**	
Forward primer	CCTGATCGCGACCGATGCGGCTGCGAAAGAAATGGTTGA
Reverse primer	TCAACCATTTCTTTCGCAGCCGCATCGGTCGCGATCAGG
***My*TIR-STING_F302R**	
Forward primer	CCTGATCGCGACCGATGCGCGTGCGAAAGAAATGGTTGA
Reverse primer	TCAACCATTTCTTTCGCACGCGCATCGGTCGCGATCAGG
